# Coexpression and Transcriptome analyses identify active Apomixis-related genes in *Paspalum notatum* leaves

**DOI:** 10.1186/s12864-020-6518-z

**Published:** 2020-01-28

**Authors:** Fernanda A. de Oliveira, Bianca B. Z. Vigna, Carla C. da Silva, Alessandra P. Fávero, Frederico P. de Matta, Ana L. S. Azevedo, Anete P. de Souza

**Affiliations:** 1 Centro de Biologia Molecular e Engenharia Genética (CBMEG), Universidade Estadual de Campinas (UNICAMP), Campinas, SP Brazil; 2 Embrapa Pecuária Sudeste, São Carlos, SP Brazil; 3 Embrapa Gado de Leite, Juiz de Fora, MG Brazil; 4 Departamento de Biologia Vegetal, Instituto de Biologia, UNICAMP, Campinas, SP Brazil

**Keywords:** Differential expression, Gene coexpression network, Apomixis, RNA sequencing, *Paspalum*

## Abstract

**Background:**

*Paspalum notatum* exhibits both sexual and apomictic cytotypes and, thus, is considered a good model for studies of apomixis because it facilitates comparative approaches. In this work, transcriptome sequencing was used to compare contrasting *P. notatum* cytotypes to identify differential expression patterns and candidate genes involved in the regulation of expression of this trait.

**Results:**

We built a comprehensive transcriptome using leaf and inflorescence from apomictic tetraploids and sexual diploids/tetraploids and a coexpression network based on pairwise correlations between transcript expression profiles. We identified genes exclusively expressed in each cytotype and genes differentially expressed between pairs of cytotypes. Gene Ontology enrichment analyses were performed to better interpret the data. We de novo assembled 114,306 reference transcripts. In total, 536 candidate genes possibly associated with apomixis were detected through statistical analyses of the differential expression data, and several interacting genes potentially linked to the apomixis-controlling region, genes that have already been reported in the literature, and their neighbors were transcriptionally related in the coexpression network.

**Conclusions:**

Apomixis is a highly desirable trait in modern agriculture due to the maintenance of the characteristics of the mother plant in the progeny. The reference transcriptome, candidate genes and their coexpression network identified in this work represent rich resources for future grass breeding programs.

## Background

RNA sequencing (RNA-seq) is the most effective method for simultaneously predicting new transcripts and identifying differentially expressed genes among distinct tissues, genotypes, abiotic conditions and developmental stages [1]. Conversely, considering the large amount of data generated from RNA-seq, new approaches that efficiently extract meaningful associations from highly multivariate datasets are needed [2]. Transcriptome coexpression studies can show how complex phenotypes depend on the activity of coordinated batteries of genes [3]. Therefore, the construction of coexpression networks based on gene expression data using correlation metrics provides valuable information regarding alterations in biological systems in response to differential gene expression patterns [2, 4].

The center of origin for the *Paspalum* genus is tropical South America, but centers of diversity have been recognized in the Brazilian cerrados and the campos of Argentina, Uruguay and Southern Brazil [5]. *P. notatum* Flüggé, also known as bahiagrass, exhibits numerous characteristics that make it an interesting system for studies investigating apomixes [6]. *P. notatum* is considered an agamic complex that includes different ploidy levels and reproductive modes, among which diploids (2n = 2 × =20) are sexual and polyploids (3×, 4×, 5×, and 6×) are pseudogamous apomicts [7]; no 4× sexual cytotypes have been found in nature [8]. Apomixis is a mode of asexual reproduction through seeds in which plant offspring are genetically identical to the female parent [9]. The inheritance of apomixis is controlled by a single complex dominant locus, i.e., epigenetically controlled parthenogenesis [10], with the capacity to form endosperm with a ratio of 4:1 (maternal:paternal) [6]. The apomixis-controlling region (ACR) in *Paspalum* is smaller than those of other apomictic systems [11] and does not demonstrate recombination, and a relatively narrow region linked to apomixis is conserved among distinct species [12, 13]. Comparative mapping of the ACR has shown synteny with a portion of the long arm of chromosome 12 in rice in *P. simplex*, *P. malacophyllum* and *P. procurrens* [12–14]. In *P. notatum*, chromosomal rearrangements have been reported in regions corresponding to rice chromosomes 2 and 12 [12, 15–17].

Apomixis can be classified in the following two ways: sporophytic, which occurs when the embryo develops from an ovule’s somatic cell through numerous mitotic divisions, and gametophytic, in which a chromosomally unreduced embryo sac can be formed from either the megaspore mother cell (diplospory) or a nearby nucellar cell (apospory) without fertilization (parthenogenesis) through a process termed apomeiosis. Endosperm formation is required for the production of viable seeds [18]. Apomixis is widely distributed among angiosperms, among which Poaceae represents the family with the largest number of apomictic genera, with the 47 apomictic species of *Paspalum* standing out in particular [6, 19]. Due to the polyploid nature of apomicts, genetic and genomic analyses are challenging.

Polyploidization is known to cause immediate and extensive genomic changes, including sequence rearrangements and/or elimination, changes in DNA methylation and loss of balance in gene expression [11]. Apomixis is frequently correlated with polyploidy and might have arisen through deregulation of the sexual developmental pathway due to an increase in the number of genomic complements through a mechanism regulated by genetic and epigenetic components [20, 21].

Apomixis has potential significance for agriculture by allowing the maintenance of heterosis in hybrid progeny. An understanding of the genomic structure of the apomictic locus is likely a prerequisite for the manipulation of the sexual pathway in model plants or economically important crops. The discovery of key genes associated with the components of apomixis and the isolation of sequence candidates have already been reported in *Paspalum* [18, 20, 22–34]*.* However, insight into the genetic mechanisms underlying asexual reproduction in natural apomicts is still needed for the development of a stable and universal system applicable to breeding programs [32]. In addition, given the complexity of this trait, an understanding of the genomic structure of the apomictic locus is likely to be an essential prerequisite for manipulation of the sexual pathway in model plants or economically important crops [35]. Nevertheless, the suppression of recombination events around the ACR limits direct genetic strategies to isolate the mechanism triggering apospory [25]. In this context, reverse genetics approaches have garnered extreme interest in the study of apomixis to identify and validate genes that are differentially expressed in apomicts and to conduct more meticulous investigations aiming to detect their positions and functions [25, 33].

The objective of this study was to use RNA-seq technology to obtain a transcriptome and comprehensively analyze the gene expression profiles of leaves and florets from apomictic and sexual genotypes with different ploidy levels in *P. notatum*. Our study revealed differentially expressed genes among the genotypes, and through the presentation of a genetic correlation network obtained by examining the de novo transcriptome, we investigate the main biological processes of genes potentially linked to the ACR. Inferences regarding candidate genes involved in the regulation of the expression of apomixis in *P. notatum* are also discussed. These candidate genes may be used to further explore and clarify the mechanisms regulating apomixis in forage grasses.

## Results

### DNA content and mode of reproduction of *P. notatum* accessions

The cytoembryological analysis (Additional file 1: Figure S1) confirmed the expected mode of reproduction of the accessions based on the literature (Tables 1 and 2). The 2C DNA content of all plants from accessions BGP_22 and BGP_306 was half the 2C DNA content from all plants from BGP_30, BGP_34, BGP_115, and BGP_216. Therefore, all evaluated plants had DNA contents compatible with the described ploidy levels of their respective accessions in the literature (Tables 1 and 2).

**Table 1:** *Paspalum notatum* accessions evaluated in this study

BGP code^a^	Former code	Origin	Collector^b^	Chromosome number
306	BRA-024236	Candói (PR), Brazil	VLiGu 14,829	2n = 2 × =20 [36]
22	BRA-006173	Bagé (RS), Brazil	VGnMaBd 9607	2n = 2 × =20 [37]
216	BRA-019470	Corrientes, Argentina	Q 3664	2n = 4 × =40 [38]
30	BRA-006467	Alegrete (RS), Brazil	VMrFrLw 9747	2n = 4 × =40 [39]
34	BRA-006513	Uruguaiana (RS), Brazil	VMrFrLw9782	2n = 4 × =40 [39]
115	BRA-010006	Laguna (SC), Brazil	VDBdSv 10,137	2n = 4 × =40 [39]

^a^BGP: internal code of germplasm bank of *Paspalum*

^b^Collectors: Bd, I. I. Boldrini; D, M. Dall’Agnol; Fr, J. M. O. Freitas; Gn, J. O. N. Gonçalves; Gu, A. Guglieri; Li, L. Essi; Lw, H. M. Longhi-Wagner; Ma, M. C. Assis; Mr., C. O. C. Moraes; Q, C. L. Quarín; Sv, G. P. da Silva; V, J. F. M. Valls; LAT, latitude (decimal degrees); LONG, longitude (decimal degrees)

**Table 2:** DNA content and modes of reproduction of the bahiagrass accessions evaluated in this study

Accession (BGP code)	DNA content				Mode of reproduction				
	Plants	2C (pg)	Δ2C among plants	Accession mean 2C (pg)	% meiotic embryo sacs	% aposporic embryo sacs	% sterile embryo sacs	Number of ovaries analyzed	Classification
30	BGP30_1^a^	3,2	0,4	3,2	38	56	6	100	Facultative apomixis
	BGP30_2^a^	3,1							
	BGP30_3	2,9							
	BGP30_4	2,8							
	BGP30_5^a^,^c^	3							
34	BGP34_1^a^,^b^,^c^	2,9	0,5	2,8	20	80	0	100	Facultative apomixis
	BGP34_2	2,9							
	BGP34_3^a^	2,5							
	BGP34_4	2,7							
	BGP34_5^a^	3							
115	BGP115_1	2,7	0,3	2,68	0	95	5	100	Obligate apomixis
	BGP115_2^a^,^c^	2,9							
	BGP115_3^a^	2,6							
	BGP115_4	2,6							
	BGP115_5^a^	2,7							
216	BGP216_1^a^,^c^	2,6	0,6	2,82	100	0	0	300	Sexual
	BGP216_2^a^	2,7							
	BGP216_3	2,9							
	BGP216_4	3,2							
	BGP216_5^a^	2,7							
22	BGP22_1	1,5	0,3	1,52	100	0	0	300	Sexual
	BGP22_2	1,7							
	BGP22_3^a^,^b^,^c^	1,4							
	BGP22_4^a^	1,4							
	BGP22_5^a^	1,5							
306	BGP306_1^a^,^c^	1,5	0,1	1,44	100	0	0	300	Sexual
	BGP306_2	1,5							
	BGP306_3	1,4							
	BGP306_4^a^	1,4							
	BGP306_5^a^	1,4							

^a^Indicates plants with foliar RNA evaluated

^b^indicates plants with inflorescence RNA evaluated

^c^indicates plants with DNA evaluated

### RNA-seq analysis and de novo transcriptome assembly

In the present study, 49.0 Gb of data were obtained, of which 87% of the reads sequenced had a Phred quality score > 30. Floret and leaf tissues were used to assemble a *P. notatum* reference transcriptome based on 313,198,097 high-quality 72-bp paired-end reads. In total, 203,808 transcripts were assembled, including a set of 114,306 nonredundant transcripts (56.08% of all transcripts) (Additional file 2: Table S1), with an average length of 750.51 bp, an N50 of 906 bp and a GC percentage of 47.60%. According to the length distribution of the nonredundant transcripts, 21,585 (18.88%) transcripts were longer than 1 kb, a size range that commonly confers a high annotation rate. More than half of all annotated transcripts were > 500 bp in length (Additional file 3: Figure S2).

Bowtie aligner mapped 94.39% of the sequenced reads onto the assembled transcripts, of which 88.79% were concordantly mapped paired ends. Considering the nonredundant transcript set, 73.78% of the sequenced reads were mapped (66.65% paired reads aligned concordantly). Of these reads, 82.97 and 83.48% of the sequencing reads belonged to sexual and apomictic samples, respectively, showing similar representation within the transcriptome. The BUSCO analysis included 956 conserved single-copy plant orthologs; the transcriptome showed high assembly completeness with 664 (69%) complete sequences (532 as a single copy and 132 as multiple copies) and 169 (17%) fragmented sequences. One hundred and twenty-three (12%) BUSCO plant orthologs were not identified in the reference *P. notatum* transcriptome.

### Transcriptome annotation

Among the 114,306 transcripts, 51,939 (45.44%) were similar to proteins from the NCBI Nr, 34,750 (30.40%) were similar to proteins from UniProtKB/Swiss-Prot, and 54,568 (47.74%) were similar to proteins from Phytozome. Overall, 21,537 (41.47%) transcripts were assigned to 4614 GO terms in the following categories: 2510 biological process, 1486 molecular function, and 618 cellular components. KEGG annotation was possible for 9221 transcripts belonging to 129 pathways, and the most represented was the Purine Metabolism Pathway (1079 members), followed by Metabolism of Thiamine (907 members) and Starch and Sucrose Metabolism (336 members). Overall, 33,873 transcripts remained without hits after searching against all protein databases; among these transcripts, open reading frames (ORFs) were found in 15,107 transcripts, and 2347 transcripts were complete. The sequences that showed ORFs without annotation require further investigation since these sequences may represent genes that have not yet been described and possibly new genes unique to *P. notatum*.

### Expression levels of genes and identification of DEGs

**Fig. 1:**
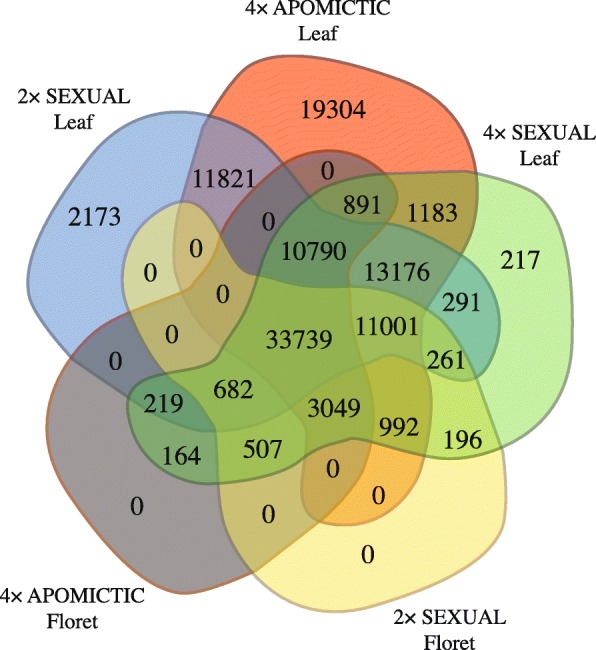
Venn diagram showing the distribution of the *P. notatum* transcripts. Expression level of transcripts from two tissues (floret and leaf) from each phenotypic class (sexual diploid, sexual tetraploid, and apomictic tetraploid)

A comparison of the transcript expression levels of the sequenced samples between the two tissues revealed genes that were expressed in only the genotypes of a phenotypic class (Fig. 1). These transcripts were considered “specific” to that class. Thus, 19,304 specific transcripts were found exclusively in the 4X apo group, 2173 specific transcripts were found exclusively in the 2X sex group, and 217 specific transcripts were found exclusively in the 4X sex group. There were no specific transcripts expressed in the florets, as all transcripts present were also expressed in the leaves (Fig. 1).

In the differential gene expression analyses, we considered only the leaf samples that contained the representative transcripts from the transcriptome assembly and examined three biological replicates (clones) per genotype. However, within the same phenotypic class, different expression patterns were observed among the genotypes, mainly in the 4X apo samples (Additional file 4: Figure S3). Thus, to reduce the effects of the genotype-based differences in expression, we performed a two-step pipeline. First, we selected transcripts that were evenly expressed within the phenotypic classes. A total of 72,318 transcripts were equally expressed among all 4X apo genotypes, and 47,069 transcripts were equally expressed in the 2X sex genotypes. This initial analysis was not necessary for 4X sex because only one genotype was analyzed. From these transcripts, we selected 28,969 transcripts that were expressed in all three phenotypic classes. Second, the pairwise comparisons of the 28,969 transcripts enabled the identification of 2072 DEGs between 2X sex and 4X apo, including 772 and 1173 overexpressed transcripts, respectively. There were 1173 DEGs between 2X sex and 4X sex, including 308 and 798 overexpressed transcripts, respectively, and 1317 genes were identified as DEGs between 4X apo and 4X sex, including 618 and 661 overexpressed transcripts, respectively. Subsequently, we inferred the enriched functions of the identified DEGs that were over- or underexpressed (Additional file 5: Figure S4, Additional file 6: Figure S5 and Additional file 7: Figure S6).

**Fig. 2:**
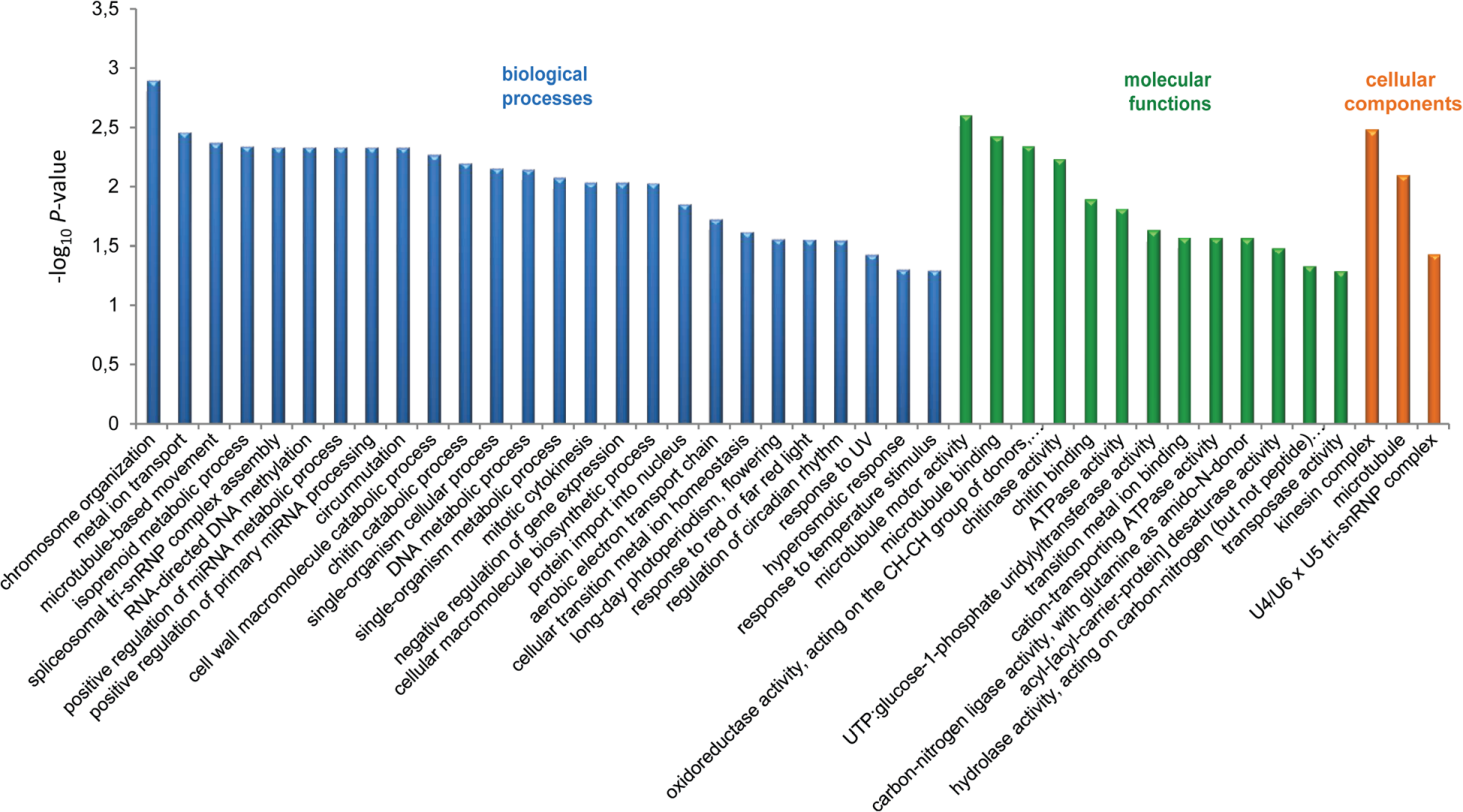
Enriched ontology terms of 510 transcripts consistently differentially expressed in the *P. notatum* transcriptome between the 4X apomictic and 2X sexual samples and between the 4X apomictic and 4X sexual samples

Among the identified DEGs, we isolated the same 510 transcripts that were differentially expressed between the 4X apomictic and the 2X sexual samples and between the 4X apomictic and 4X sexual samples, regardless of the ploidy of the sexual samples. The GO terms enriched in this set belonged to 26 biological processes (BP), 13 molecular functions (MF), and three cellular components (CC) (Fig. 2). We considered these transcripts to be potentially involved in the determination of the reproductive mode.

### qRT-PCR validation of RNA-seq data

We validated the in silico analyzes using a subset of the DEGs between the apomictic and sexual samples through an independent technique, i.e., qRT-PCR. Out of the eight genes selected for internal control, three showed nonspecific DNA amplification and were discarded, and five were successfully amplified, showed desirable expression stability and coefficients of variance among the samples, showing M values < 0.5 and CV values < 0.25 (Additional file 8: Table S2). Of these genes, one gene was selected as an internal control for qRT-PCR verification of the 2X sex vs. 4X apo analyses (01RefGen-*Pnot*), and one gene was selected for verification of the 4X apo vs. 4X sex analyses (04RefGen-*Pnot*) due to their expression stability between the samples of each group. Different internal controls were used for the different groups of samples because we opted for increased accuracy in the analyses instead of risking a biased result using the same control for the sample groups. The transcriptionally stable genes we adopted in our qRT-PCR may also serve as reference genes for other studies.

The 18 primer pairs designed for the target DEGs, were initially evaluated through PCR using genomic DNA as a template. Eight did not amplify or presented nonspecific amplification and were discarded. Two primer pairs were not evaluated in qRT-PCR but showed interesting results in terms of DNA amplification. The first was designed from a transcript that was exclusively expressed in apomict samples in RNA-seq analyses (i.e., showing a zero expression value in the sexual samples). This transcript did not amplify using genomic DNA from sexual samples as PCR templates. For the second primer pair, although genomic DNA amplification occurred in all samples, when complementary DNA (cDNA) was used as a PCR template, only the apomictic samples presented amplicons. The expression level of this transcript estimated from RNA-seq of the same samples was very low in the 2X sex samples. These results suggest that the first transcript, which is expressed only in apomictic cytotypes, is exclusive to the genomes of these samples, whereas the second transcript was poorly expressed or silenced in sexual samples.

Finally, eight primer pairs successfully produced amplicons and were used for qRT-PCR validation (Additional file 9: Table S3), revealing results that were consistent with those obtained through RNA-seq (Additional file 10: Figure S7 and Additional file 11: Figure S8).

### Detailed search for potential apomixis genes

We detected 1612 transcripts in the *P. notatum* reference transcriptome that showed high similarity to the putative ACR region syntenic to rice chromosomes 2 and 12. Of these transcripts, 1356 transcripts aligned to the correspondent rice chromosome 2, and 256 transcripts aligned to rice chromosome 12. Interestingly, 40 transcripts were a part of the set identified as apomictic specific; nine transcripts were specific to the 2X sex samples, and six transcripts were specific to the 4X sex samples. Moreover, considering the analyses of the DEGs, 16 putative ACR transcripts were differentially expressed between 2X sex and 4X apo; 20 DEGs were observed between 4X apo and 4X sex; and 17 DEGs were observed between 2X sex and 4X sex. However, in total, there were 89 different transcripts, because some were repeated as differentially expressed in more than one pairwise comparison.

These findings, especially this 89-transcript set (Additional file 12: Table S4), represent valuable genes that deserve to be carefully investigated to determine their roles in apomixis and whether they are effective within the ACR in *P. notatum*.

The BLAST results shown in Additional file 13: Table S5 highlight the similarity scores of the *P. notatum* transcripts to *Paspalum* sequences previously associated with apomixis, revealing that our transcriptome contains genes already found to be involved in asexual reproductive development.

### Transcript coexpression network

**Fig. 3:**
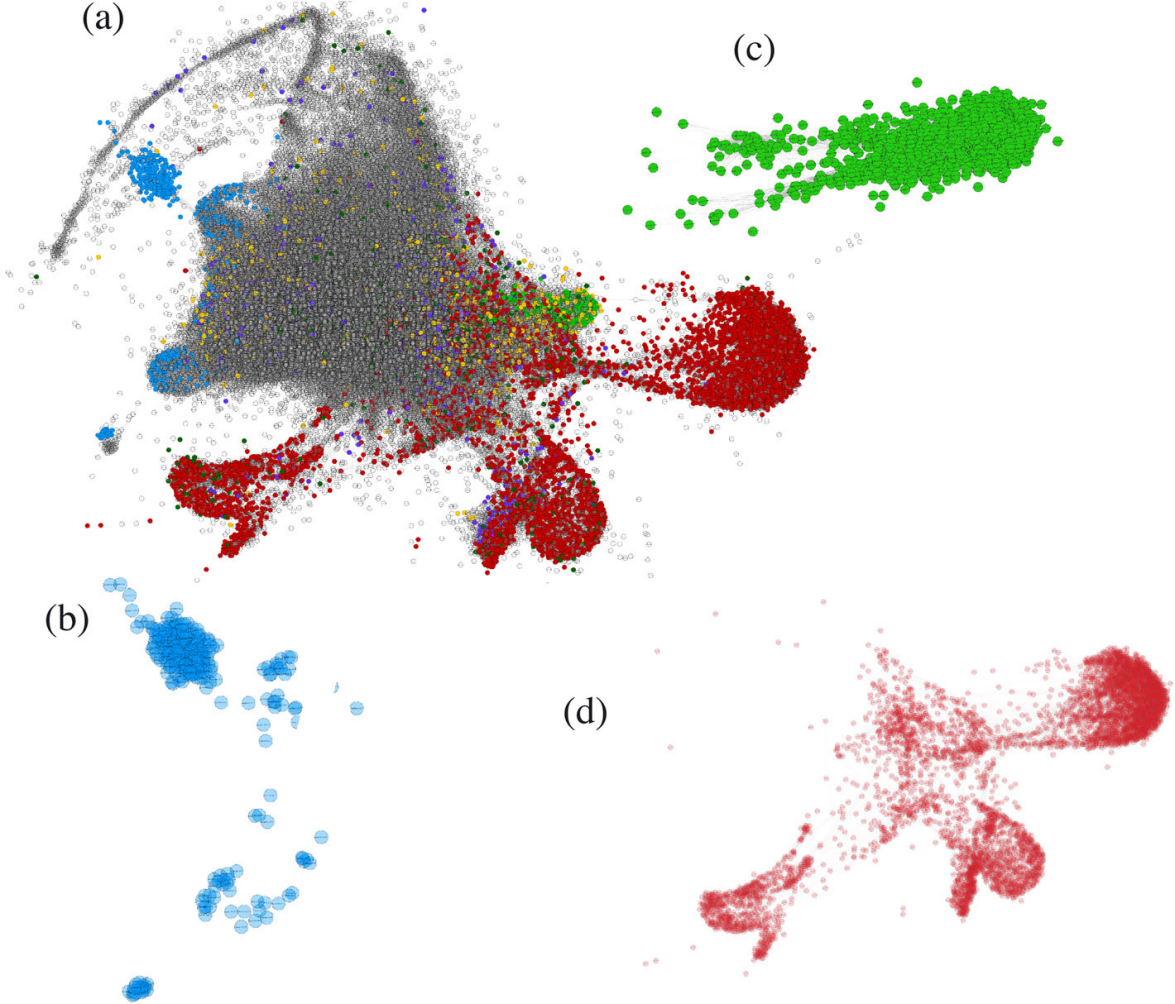
Coexpression network of *Paspalum notatum*. **a** Coexpression network of all analyzed transcripts; the more centralized genes shown in light gray are common to all genotypes analyzed with nondifferential expression. Node color denotes the differentially expressed transcripts among the phenotypic classes as follows: purple (2X sex vs. 4X apo), yellow (2X sex vs. 4X sex), and dark green (4X apo vs. 4X sex). Networks of exclusively expressed transcripts in each phenotypic class are highlighted as follows: (**b**) transcripts specific to sexual diploids (blue); (**c**) transcripts specific to sexual tetraploids (green); and (**d**) transcripts specific to apomictic tetraploids (red). Edges denote interaction strength. Circular nodes represent transcripts

We identified 879,481 connections among the 53,262 transcripts from the RNA-seq data (Fig. 3). The transcripts were grouped into 642 clusters according to their correlated pattern of expression level. In the network, the specific transcripts in each phenotypic class tended to form coexpression modules (Fig. 3b-d). Despite this subdivision, the relevant biological relationships of these transcripts with all remaining transcripts can be recovered in a fully integrated network.

**Fig. 4:**
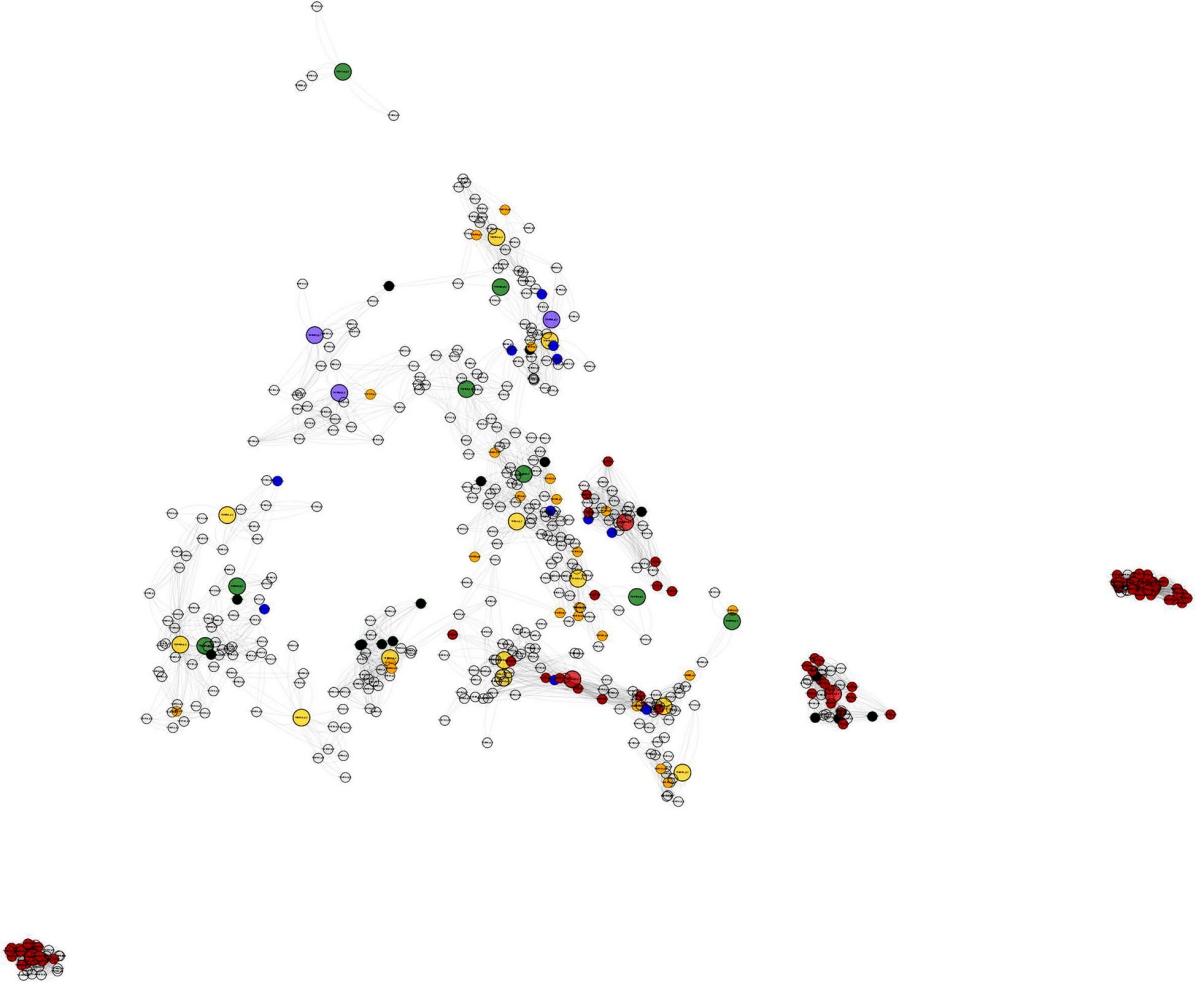
Gene expression subnetwork of 536 *Paspalum notatum* transcripts. The 89 differentially expressed and/or specific putative ACR transcripts are presented in this subnetwork along with their first neighbors. The color patterns are the same as those used in the complete coexpression network shown in Fig. 3

**Fig. 5:**
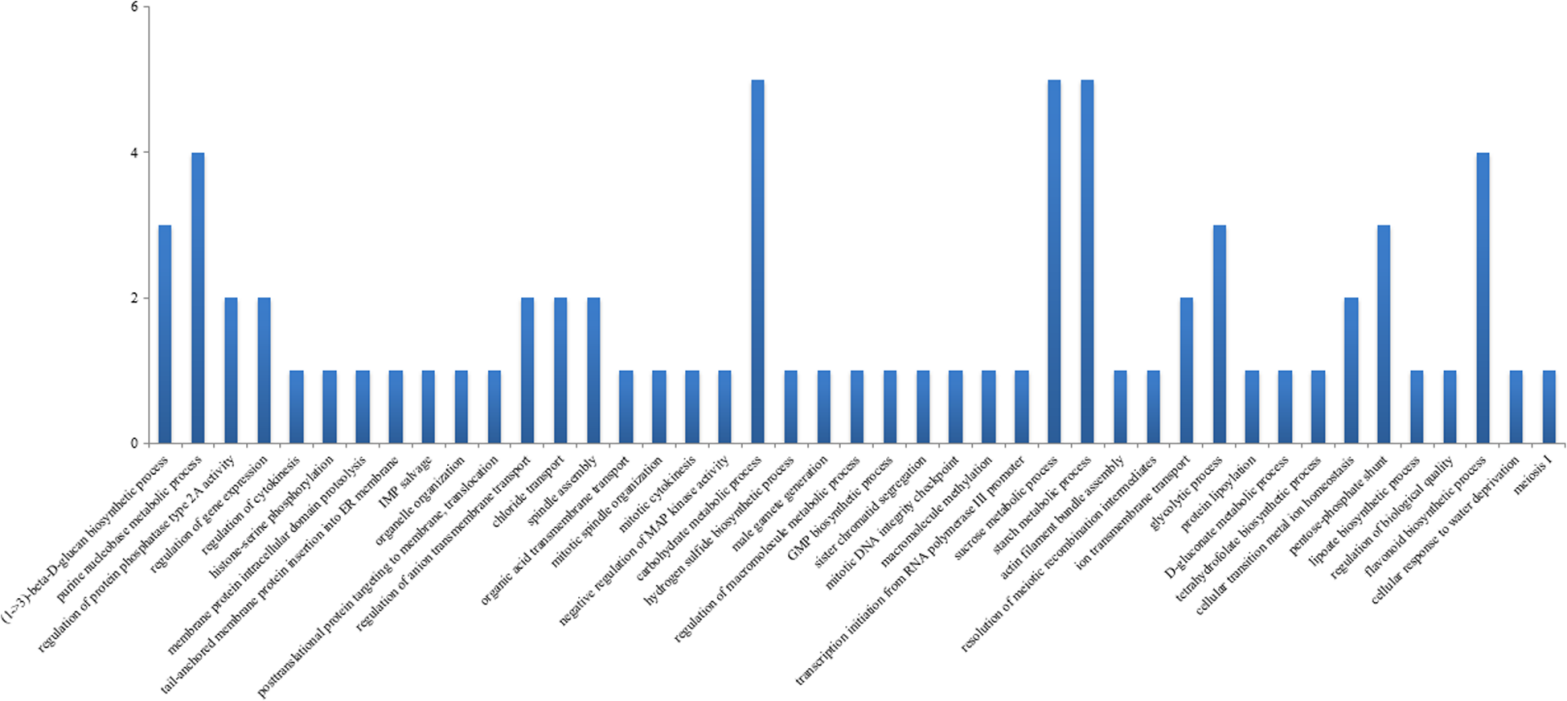
Enriched biological processes in the 536 transcripts in the gene expression subnetwork

To identify the direct correlations among the 89 differentially expressed and/or specific putative ACR genes and their first neighbors, we retrieved a subnetwork composed of 536 strongly correlated transcripts (Fig. 4). We used a GO enrichment analysis to summarize the main functions of this transcript set, which included 43 BP, 31 MF and 17 CC (Fig. 5).

## Discussion

We constructed a *P. notatum* reference transcriptome using RNA-seq to investigate the gene expression changes in apomictic and sexual genotypes. This analysis enabled the assembly of a nonredundant transcriptome containing 114,306 transcripts from florets and leaves from six distinct genotypes with different ploidy levels and reproductive modes. Robust metrics indicated that the transcriptome was a quality assembly with a high degree of integrity.

Although we did not sequence inflorescence samples at different developmental stages to search for candidate apomixis-linked genes, we isolated expression patterns of phenotypic classes, aiming both at removing transcripts whose expression could be related to the effect of genotype and at identifying transcripts expressed in all genotypes within the same phenotypic class. Differential expression analyses of leaf samples from sexual and apomictic cytotypes allowed us to identify DEGs that may be representative of reproductive mode and not dependent on ploidy level.

The GO classification of the assembled transcriptome into 4614 known terms was used to perform functional enrichment analyses. Based on the enriched GO terms of a set of 510 DEGs detected between apomictic and sexual, we emphasize “positive regulation of miRNA metabolic process (GO:2000630)” and “regulation of primary miRNA processing (GO:2000636)”, both of which activate or increase the frequency, rate, or extent of miRNA production. Furthermore, miRNA is directly related to “DNA metabolic process (GO:0006259)”, which decreases the rate of gene expression (negative regulation of gene expression - GO:0010629) through an epigenetic RNA-based gene silencing process (RNA-directed DNA methylation - GO:0080188). Five transcripts were mainly involved in these processes and showed higher expression levels in the apomict samples; these transcripts correspond to a pseudoARR-B transcription factor; pre-mRNA splicing factor, putative (*Ricinus communis*); hypothetical protein SORBIDRAFT_05g016770; uncharacterized protein, LOC100501330 (*Zea mays*); and an uncharacterized protein LOC103641690. The significant enrichment of terms associated with the regulation of gene expression by epigenetic silencing among the transcripts was higher in the apomictic samples compared with that in the sexual samples of bahiagrass. This result is consistent with the growing body of evidence that suggests that apomixis arises from the deregulation of the sexual pathway, where epigenetic mechanisms play a significant role in at least some elements of apomictic development [6, 40].

These 510 DEGs along with the 19,304 transcripts that were exclusively expressed in the apomictic samples represent a set of candidate genes that deserves further investigation. In apospory, gene expression occurs at specific stages, such as apomeiosis, parthenogenesis, and endosperm development. The increased or decreased expression of some genes during these stages may hinder the analysis of differential expression between sexual and apomictic genotypes [33, 41]. Nonetheless, we believe that the identification of DEGs as performed here allowed for the detection of gene expression patterns related to characteristics shared among genotypes independently of physiological or developmental particularities. Thus, using selected transcripts with a detectable expression pattern among all genotypes of each phenotypic class may have minimized that influence. Simultaneously, our approach increased the potential to discover candidate genes involved in many steps of the apomictic process. It is important to emphasize that the group of apomictic genotypes consisted of two facultative apomictic genotypes and one obligatory apomictic genotype and that all selected expressed genes were expressed in the obligatory apomictic sample. However, some amount of background noise might be present. Thus, future reverse genetic experiments based on qRT-PCR and in situ hybridization could be useful for identifying the role of specific genes throughout the apomixis process.

The size of the ACR in *Paspalum* is smaller than those in other apomictic systems [6], but the lack of recombination hinders the use of map-based cloning approaches in studies investigating this region [25]. Thus, the discovery of candidate genes that are potentially located within this region is extremely valuable for an understanding of the complex regulatory network of gene–gene interactions. Thus, DEGs could be used for the future manipulation of the trait, which has outstanding importance in agricultural biotechnology.

Furthermore, the recovery of the previously published sequences demonstrates the representative and informative nature of the assembled transcriptomes and enhances our understanding of global RNA expression. In *Paspalum,* the first attempt to understand the molecular basis of apomixis was performed by Pessino et al. [23]. This study led to the identification of three small sequences expressed during early megagametophyte development in apomictic plants of *P. notatum*. Here, we recovered the reported sequences as a single representative transcript (Additional file 13: Table S5) that is similar to a kinesin motor protein involved in microtubule-based movement. This protein has been reported to be differentially expressed between apomictic and sexual plants [22, 25, 42], and the same pattern was observed in the transcripts assembled in this work.

Subsequently, several other candidate genes have been reported using *P. notatum* and *P. simplex* [20, 23–25], and the first study using RNA-seq was performed in bahiagrass based on expression profiling of apomictic and sexual flowers [33]. Some candidate genes have been investigated in more detail [28, 30–32]. However, many questions remain unanswered, and further research is needed to define the relationships among the structure, position, and function of known apomixis-linked genes [6].

Based on a set of genes selected through differential expression patterns, we recovered previously published sequences in the *P. notatum* transcriptome. n20gap-1, which is one of these previously reported genes in the literature, is a Lorelei-like *P. notatum* gene [25, 28] that encodes a GPI-anchored protein that supposedly plays a role in the final stages of the apomixis developmental cascade. Laspina et al. [25] reported that this sequence is linked to the chromosomal locus governing apospory at a genetic distance of 22 cM. Here, one transcript similar to n20gap-1 was present in the set of transcripts potentially integrated within the ACR, corresponding to rice chromosome 2. Additionally, we found differentially expressed transcripts that aligned with the sequences characterized by Podio et al. [30], corresponding to *Somatic Embryogenesis Receptor-Like Kinase* (*SERK*), in addition to numerous other transcripts with the same annotation. The candidate SERK-like genes play crucial roles in somatic embryogenesis in angiosperms and have been reported to be related to apomixis. We also identified *P. notatum* transcripts that showed similarity to the PnTgs1-like gene that encodes a trimethylguanosine synthase-like protein, which plays a fundamental role in nucellar cell fate, and its diminished expression has been reported to correlate with the initiation of the apomictic pathway [31]. Additionally, two transcripts were similar to the sequence of *Ps*ORC3, which seems to play an active role in the mechanisms repressing sexuality in apomictic *P. simplex* [32].

Functionally related genes tend to be transcriptionally coordinated [43]. Therefore, the construction of a coexpression network provided a powerful resource for the identification of transcripts that are coregulated with specific genes and DEGs despite the lack of detectable differences in expression among the samples. The subnetwork containing 536 coexpressed transcripts possibly associated with the expression of apomixis, is an example of a valuable approach to finding likely biological relationships among genes of interest using transcriptomic approaches. By starting on a smaller scale of transcripts that are DEGs and/or specific genes possibly integrated into the ACR and combining information by adding their nearest neighbor genes, we obtained a broader view of the processes involved in the regulation of candidate genes. The significantly enriched BP, such as “male gamete generation (GO: 0048232)”, “sister chromatid segregation (GO: 0000819)”, “meiosis I (GO: 0007127)”, “resolution of meiotic recombination intermediates (GO: 0000712)”, “regulation of cytokinesis (GO: 0032465)”, “mitotic cytokinesis (GO: 0000281)”, “spindle assembly (GO: 0051225)”, “mitotic spindle organization (GO: 0007052)”, and “mitotic DNA integrity checkpoint (GO: 0044774)”, were associated with plant reproduction. Additionally, the subnetwork was enriched in epigenetic processes, such as “macromolecule methylation (GO: 0043414)”, “histone-serine phosphorylation (GO: 0035404)”, “regulation of protein phosphatase type 2A activity (GO: 0034047)”, and “negative regulation of MAP kinase activity (GO: 0043407)”. The transcripts in the subnetwork were associated with reproductive processes and the regulation of epigenetic changes by modulating histones. It is important to note that the MAP kinase activity was negatively regulated in this work, and this gene family also has been reported as being related to the apomictic reproduction. The mitogen-activated protein 3-kinase (MAP3K) gene family has been identified as differentially expressed between apomictic and sexual flowers of *P. notatum* [25] and flowers of *P. simplex* [20] and might play a role in the parthenogenetic development of embryos in both species [6]. A gene essential for the formation of unreduced embryo sacs in *P. notatum* was identified from the characterization of MAP3K retrieved from transcriptomic surveys [44]. The information presented in the gene coexpression network in this study may highlight candidate genes for the study of apomixis in this species.

In this study, we applied RNA-seq technology and bioinformatics methods to assemble a useful transcriptome and identify differences between apomictic and sexual reproduction. Our results reveal DEGs and genes exclusively expressed in apomicts or sexuals that are potentially associated with the ACR genomic region, including the apo locus. The gene coexpression network developed by this work represents an advanced and promising genetic technique for studies in *P. notatum*. In this work, the technique enabled a more comprehensive analysis of the transcripts obtained from our *P. notatum* transcriptome that are potentially involved in the regulation of apomixis. The validation of genes in this set of candidates may provide valuable insight to enhance our understanding of apospory. Moreover, these genes may be considered in screening for molecular markers linked to apomixis in *P. notatum*, which will be crucial for boosting breeding programs for apomictic forage grasses.

## Methods

### Plant materials and RNA extraction

Six accessions of *P. notatum* belonging to the Germplasm Bank of *Paspalum* and maintained by Embrapa Pecuária Sudeste, São Carlos, SP, Brazil (22° 01′S and 47°54′ W; 856 m above sea level) (Table 1) were chosen based on their origins (different ecotypes), genetic dissimilarity [45], ploidy levels and reproduction modes. The Q3664 (BGP 216) accession is a hybrid from a cross between a sexual colchicine-induced tetraploid (PT-2) and a white-stigma apomictic tetraploid and is characterized as a facultative apomictic accession with a high level of sexuality (> 70%) [46].

Five clones of each accession were planted in 8 L pots, filled with 1:1 soil/vermiculite and grown in a greenhouse under identical environmental conditions. The climate is humid subtropical (according to the Köppen-Geiger classification system), with annual average low and high temperatures of 15.3 and 27.0 °C and a total rainfall of 1422.8 mm occurring mainly during the spring and summer seasons [47]. Young leaf samples from three clones of each accession were collected during the summer (December). One clone of the accessions BGP34 (apomictic tetraploid) and BGP22 (sexual diploid) presented inflorescences, which were collected. All leaf and floret samples were immediately placed in liquid nitrogen and subsequently stored at − 80 °C until RNA extraction. The total RNA was isolated according to Oliveira et al. [48]. RNA integrity was assessed in a 1% agarose denaturing gel and quantified using a NanoVue Plus spectrophotometer (GE Healthcare Life Sciences, Little Chalfont, UK).

### Mode of reproduction through cytoembryological analysis

The mode of reproduction of the plants was confirmed using the embryo sac clarification method proposed by Young et al. [49], with minor modifications. Inflorescences at anthesis (when the embryo sac is fully developed) were fixed in FAA (95% ethanol, distilled water, glacial acetic acid, and formalin 40% at 40:13:3:3 v/v) for 24 h at room temperature. The embryo sacs were clarified as followed: 85% ethanol; absolute ethanol; ethanol:methyl salicylate (1:1); ethanol:methyl salicylate (1:3); and 100% methyl salicylate (two times). The samples remained in each solution for 24 h. Then, 100 to 300 embryo sacs per accession were evaluated under an Axiophot microscope (Carl Zeiss, Jena, Germany) using the differential interference contrast (DIC) microscopy technique.

### DNA content estimation through flow cytometry

Flow cytometry analyses were performed to confirm the exact ploidy level (Table 1). Approximately 5 mg of young leaf tissue from each clone were used (Table 2). *Pisum sativum* cv. Ctirad (2C = 9.09 pg) was used as an internal control [50]. The samples were triturated in Petri dishes containing 800 μL of LB01 buffer (0.45425 g TRIS, 0.186125 g NaEDTA, 0.0435 g-spermine, 0.29225 g NaCl, 1.491 g KCl, and 250 μL of Triton X-100 in 250 mL of distilled water, 7.5 pH, 0.11% v/v β-mercaptoethanol) [51]. The nuclear suspension was filtered through a 40-μm mesh and incubated at room temperature for 5 min, followed by the addition of 25 μL of propidium iodide and 25 μL of RNase. For each sample, at least 10,000 nuclei were analyzed with a FACSCalibur flow cytometer (Becton Dickinson, New Jersey, USA). Histograms were generated using Cell Quest software and analyzed using Flowing Software (available at http://www.flowingsoftware.com). The nuclear DNA index (pg) was estimated based on the value of the G1 peak. We calculated the mean value of C per clone and accession and the difference between the highest and lowest values (Δ) observed in the replicates of each accession.

### RNA-seq library, Illumina sequencing, and data quality control

cDNA libraries were constructed from 18 leaf and two floret samples using a TruSeq RNA Sample Preparation Kit (Illumina Inc., San Diego, CA, USA) following the manufacturer’s instructions. Library quality was confirmed using the Agilent 2100 Bioanalyzer (Agilent Technologies, Palo Alto, CA, USA) and quantified via quantitative real-time PCR (qPCR) (Illumina protocol SY-930-10-10). Clustering was performed using a TruSeq Paired-End Cluster Kit on a cBot (Illumina Inc.). Paired-end sequencing was performed on an Illumina Genome Analyzer IIx Platform with TruSeq SBS 36-Cycle kits (Illumina Inc.) following the manufacturer’s specifications. RNA-seq was performed with floret libraries in two separate lanes without biological replicates. The remaining 18 leaf libraries from six different accessions (Table 1), each with three biological replicates, were distributed randomly in the flow cell with three libraries per lane.

The raw data was converted to FastQ files containing 72-bp reads. Quality control was performed using the NGS QC Toolkit v2.3.3 [52]. Initially, high-quality reads (Phred quality score ≥ 20 in at least 75% of bases) and reads with more than 60 bases were selected. Subsequently, reads were trimmed at the 3′ end for the removal of barcodes.

### Transcriptome assembly and completeness assessment

The reads from the leaves and florets were de novo assembled into a reference transcriptome with Trinity v2.0.2 [53] using default settings. The contig redundancy was minimized through the selection of the first Butterfly sequence from each Chrysalis component, which is considered the most representative contig. The reads were mapped to the transcriptome using Bowtie2 sequence aligner v2.2.5 [54]. The Benchmarking Universal Single-Copy Orthologs (BUSCO) tool, an approach assessing conserved orthologs among plant species in a set of sequences [55], was used to estimate the completeness of the transcriptome assembly.

### Transcriptome annotation

The transcripts were aligned to proteins from the NCBI nonredundant (Nr) database, UniProtKB/Swiss-Prot and Phytozome v9.0 using the BLASTX algorithm with an e-value cutoff of 1e-06. The Gene Ontology (GO) terms were retrieved using Blast2GO software. We used REVIGO [56] to summarize the GO term sets. The transcripts were assigned to metabolic pathways via the Kyoto Encyclopedia of Genes and Genomes (KEGG) database [57].

### Transcript expression levels

The expression levels were estimated using the FPKM method (expected number of fragments per kilobase of transcript sequence per million base pairs sequenced). The read counts were obtained using Bowtie v2–2.1.0 and normalized using the RSEM software (RNA-seq by expectation maximization) [58]. A Venn diagram was created using the online platform available at http://bioinformatics.psb.ugent.be/webtools/Venn/. A principal component analysis (PCA) was used to assess the expression patterns of the sequenced genotypes.

### Differential expression analysis

EBSeq [59] was used to identify the differentially expressed genes (DEGs) using the FPKM values at a false discovery rate (FDR) ≤ 0.05. Transcripts with a log2-fold change (FC) ≥ 1.5 in abundance were regarded as overexpressed. The GO enrichment analysis was carried out using the ‘goseq’ 1.24.0 Bioconductor package [60]. The *P*-values were subjected to Bonferroni correction; adjusted P-values ≤0.05 were considered enriched and summarized using REVIGO [56].

**Fig. 6:**
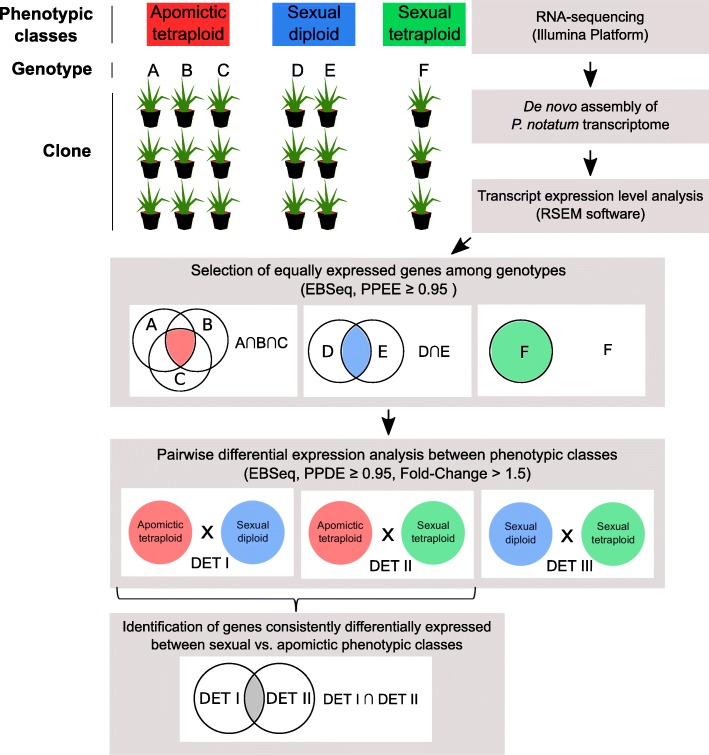
Workflow of differential expression analyses used in this study. We performed pairwise differential expression analyses of all three phenotypic classes (apomictic tetraploid “4X apo”, sexual diploid “2X sex” and sexual tetraploid “4X sex”). Each phenotypic class had different genotypes with three clones. First, the objective was to select a list of equally expressed transcripts among genotypes belonging to the same phenotypic class. EBSeq was used to estimate the pairwise posterior probabilities of a transcript being equally expressed (PPEE) among genotypes of the same phenotypic class as follows: apomictic tetraploid (A × B; A × C; B × C) and sexual diploid (D × E). Transcripts with PPEE ≥0.95 were kept for the second step of the analysis. Second, another round of pairwise differential expression analyses among phenotypic classes was performed as follows: (i) 4X apo vs. 2X sex; (ii) 4X apo vs. 4X sex; and (iii) 2X sex vs. 4X sex. The objective was to select a list of transcripts that presented pairwise posterior probabilities of being differentially expressed (PPDE ≥0.95). Additionally, we identified a list of transcripts that were consistently differentially expressed between the sexual and apomictic samples independently of the ploidy level (diploid or tetraploid)

The genotypes were grouped according to their phenotypic classes as sexual diploids (2X sex), sexual tetraploids (4X sex) and apomictic tetraploids (4X apo) and compared in pairwise differential expression analyses. A two-step pipeline was used for the identification of the DEGs (Fig. 6). In the first step, the objective was to select a list of equally expressed transcripts among genotypes belonging to the same phenotypic class. This procedure allowed the detection of gene expression patterns related to characteristics that were shared among the genotypes, independently of physiological or developmental particularities. Thus, EBSeq was used to estimate the pairwise posterior probabilities of a transcript being equally expressed between genotypes as follows: 2X sex (BGP22 and BGP306) and 4X apo (BGP30, BGP34 and BGP115); thus, a list of equally expressed transcripts (PPEE ≥0.95) among genotypes belonging to the same phenotypic class were retained for the second step of the analysis. The second step consisted of another round of pairwise differential expression analyses (PPDE) between phenotypic classes: (i) 2X sex and 4X apo; (ii) 2X sex and 4X sex; (iii) 4X apo and 4X sex.

### Quantitative reverse transcription PCR (qRT-PCR) validation

To verify the reliability and accuracy of the transcriptome data and validate the differential expression results, 18 DEGs were randomly selected for quantification through reverse transcription PCR (qRT-PCR). Eight transcripts that displayed similar expression patterns based on FPKM were selected to be evaluated as control genes. The primer sets were designed using Primer3Plus software [61]; the target amplicon size was set to 70–150 bp, with an optimal annealing temperature of 60 °C and an optimal primer length of 20 bp. All primer pairs were initially tested in regular PCRs using genomic DNA from the same samples used in RNA-seq as the template. Only primers that amplified the genomic DNA of all genotypes were used in the following qRT-PCR. Primer pairs that successfully amplified the DNA of all genotypes and showed an amplification efficiency of 90–110% and R^2^ > 0.99 were used for the relative expression analyses.

Total RNA (500 ng) was used for cDNA synthesis using the QuantiTect Reverse Transcription Kit (Qiagen Inc., Chatsworth, CA, USA). The cDNAs were diluted (1:10) in nuclease-free water, and 2 μL was used for qRT-PCR. qRT-PCR was performed using a CFX384 Real-Time PCR Detection System with iTaq Universal SYBR Green Supermix (Bio-Rad Laboratories Inc., Hercules, CA, USA) according to the manufacturer’s instructions, and the final primer concentration was 0.3 μM. The reaction conditions were 95 °C for 10 min, followed by 40 cycles of 95 °C for 30 s and 60 °C for 1 min. No-template controls for each primer pair were included, and all experiments were performed in triplicate using independent samples. The specificity of the amplicons was confirmed through analysis of the melting curve with temperatures increasing from 65 °C to 95 °C with increments of 0.5 °C. The baseline and Cq (quantification cycle) values were automatically determined, and the expression values were estimated using ΔΔCt by CFX Manager 2.1 software (Bio-Rad Laboratories, Inc., USA). The reference genes were selected according to the gene expression stability values (M < 0.5) and coefficients of variance (CV < 0.25) among the samples. Mann-Whitney U-tests were performed to estimate the statistical significance.

### Search for putative apomixis genes

A comparative analysis of *P. notatum* transcripts with their syntenic counterparts on rice chromosomes conservatively linked to apomixis in *Paspalum* species was performed [6]; we aligned all *P. notatum* transcripts to rice transcripts using BLASTN in the highlighted area of rice chromosomes 2 and 12, which were delimited by a set of molecular markers completely linked to the apospory locus. This region encompassed the C1069 and C996 markers of rice chromosome 12 and between the C560 and C932 markers of chromosome 2 [6, 12, 15–17, 62]. We also compared the transcriptome with sequences reported to be potentially associated with apomixis in *Paspalum* using BLASTN with an e-value cutoff of 1e-05.

### Coregulatory networks

For the coexpression network, we used the FPKM of all assembled transcripts from the floret and leaf tissues with a Pearson correlation coefficient cutoff of ≥0.8. Transcripts showing null values for most clones were excluded to reduce noise. The highest reciprocal rank (HRR) [63] method with a limit of 30 was used to select the strongest edges. A heuristic cluster chiseling algorithm (HCCA) was applied with three-step node vicinity networks (NVN) [63]. Cytoscape 3.4.0 [64] was used for visualization.

## Supplementary information

**Additional file 1: Figure S1.** Clarified embryo sacs visualized by differential interference contrast (DIC) microscopy from the *Paspalum notatum* accessions evaluated in this work. (A) Apomictic and (B) sexual BGP 30 embryo sacs. (C) Apomictic and (D) sexual BGP 34 embryo sacs. (E) Apomictic embryo sac from BGP 115. (F), (G) and (H) Sexual embryo sacs from BGP 306, BGP 22 and BGP 216, respectively.

**Additional file 2: Table S1.** Statistics of the assembled transcriptome of *Paspalum notatum*.

**Additional file 3: Figure S2.** Length distribution of the number of nonredundant transcripts successfully annotated. Comparison of all nonredundant transcripts and the number of transcripts annotated in the NCBI nonredundant protein database by size range.

**Additional file 4: Figure S3.** Principal component analysis (PCA) according to the FPKM values of the *P. notatum* transcriptome. PCA of leaf and floret transcriptomes of all genotypes and clones used in RNA-seq.

**Additional file 5: Figure S4.** Functional classification of enriched overexpressed DEGs in the 2X sex vs. the 4X apo comparison. Gene Ontology biological process (blue boxes), Gene Ontology cellular component (yellow boxes), and Gene Ontology molecular function (orange boxes). a) Categories enriched in overexpressed transcripts in 2X sex and b) categories enriched in overexpressed transcripts in 4X apo.

**Additional file 6: Figure S5.** Functional classification of enriched overexpressed DEGs in the 2X sex vs. 4X sex comparison. Gene Ontology biological process (blue boxes), Gene Ontology cellular component (yellow boxes), and Gene Ontology molecular function (orange boxes). a) Categories enriched in overexpressed transcripts in 2X sex and b) categories enriched in overexpressed transcripts in 4X sex.

**Additional file 7: Figure S6.** Functional classification of enriched overexpressed DEGs in the 4X apo vs. 4X sex comparison. Gene Ontology biological process (blue boxes), Gene Ontology cellular component (yellow boxes), and Gene Ontology molecular function (orange boxes). a) Categories enriched in overexpressed transcripts in 4X apo and b) categories enriched in overexpressed transcripts in 4X sex.

**Additional file 8: Table S2.** Primer sequences and amplicons of the candidate reference genes evaluated in this study.

**Additional file 9: Table S3.** Quantitative RT-PCR primer sequences.

**Additional file 10: Figure S7.** Histograms of gene expression obtained by qRT-PCR. qRT-PCR validation showing the relative expression patterns of 4 genes differentially expressed between the tetraploid apomictic “4X apo “ (red box) and diploid sexual “2 × SEX” (blue box) samples. *P < 0.05; **P < 0.01; ***P < 0.001; statistically significant differences in gene expression between phenotypic classes compared using the Mann-Whitney U-test.

**Additional file 11: Figure S8.** Histograms of gene expression obtained by qRT-PCR. qRT-PCR validation showing the relative expression patterns of 4 genes differentially expressed between the tetraploid apomictic “4X apo “ (red box) and tetraploid sexual “4X sex” (green box) samples. *P < 0.05; **P < 0.01; ***P < 0.001; statistically significant differences in gene expression between the phenotypic classes compared using the Mann-Whitney U-test.

**Additional file 12: Table S4.** Functional classification of the 89 differentially expressed and/or specific putative apomixis-controlling region (ACR) transcripts.

**Additional file 13: Table S5.** Results of the BLAST search of sequences of *P. notatum* from the literature against the assembled transcriptome.

## Data Availability

The datasets generated during the current study are available in the NCBI Short Read Archive (SRA) repository, under accession number SRP150615. This research is registered in the Ministry of the Environment (SisGen) under the number AA835F9 for the regulation of the Brazilian law number 13.123/2015. Other datasets used and/or analyzed during the current study are available from the corresponding author on reasonable request.

## Abbreviations

2X sex: Sexual diploids
4X apo: Apomictic tetraploids
4X sex: Sexual tetraploids
ACR: Apomixis-controlling region
BP: Gene ontology biological process
BUSCO: Benchmarking universal single-copy orthologs
CC: Gene ontology cellular component
cDNA: Complementary DNA
Cq: Quantification cycle
CV: Coefficients of variance
DEG: Differentially expressed gene
DIC: Differential interference contrast
FC: Fold change
FDR: False discovery rate
FPKM: Fragments per kilobase of transcript sequence per million base pairs sequenced
GO: Gene ontology
HCCA: Heuristic cluster chiseling algorithm
HRR: Highest reciprocal rank
KEGG: Kyoto encyclopedia of genes and genomes database
M: Gene expression stability
MAP3K: Mitogen-activated protein 3-kinase
MF: Gene ontology molecular function
Nr: NCBI nonredundant protein database
NVN: Node vicinity networks
ORF: Open reading frame
PCA: Principal component analysis
PPDE: Differential expression analyses
PPEE: Equally expressed transcripts
qPCR: Quantitative real-time PCR
qRT-PCR: Quantitative reverse transcription PCR
RNA-seq: RNA sequencing
RSEM: RNA-Seq by expectation maximization
SERK: Somatic embryogenesis receptor-like kinase
SRA: NCBI short read archive
