# Supplementary figures and images for: Coexpression and Transcriptome analyses identify active Apomixis-related genes in Paspalum notatum leaves
Source: BMC Genomics. 2020 Jan 28;21:78. doi: 10.1186/s12864-020-6518-z (PMC6986084; doi:10.1186/s12864-020-6518-z)

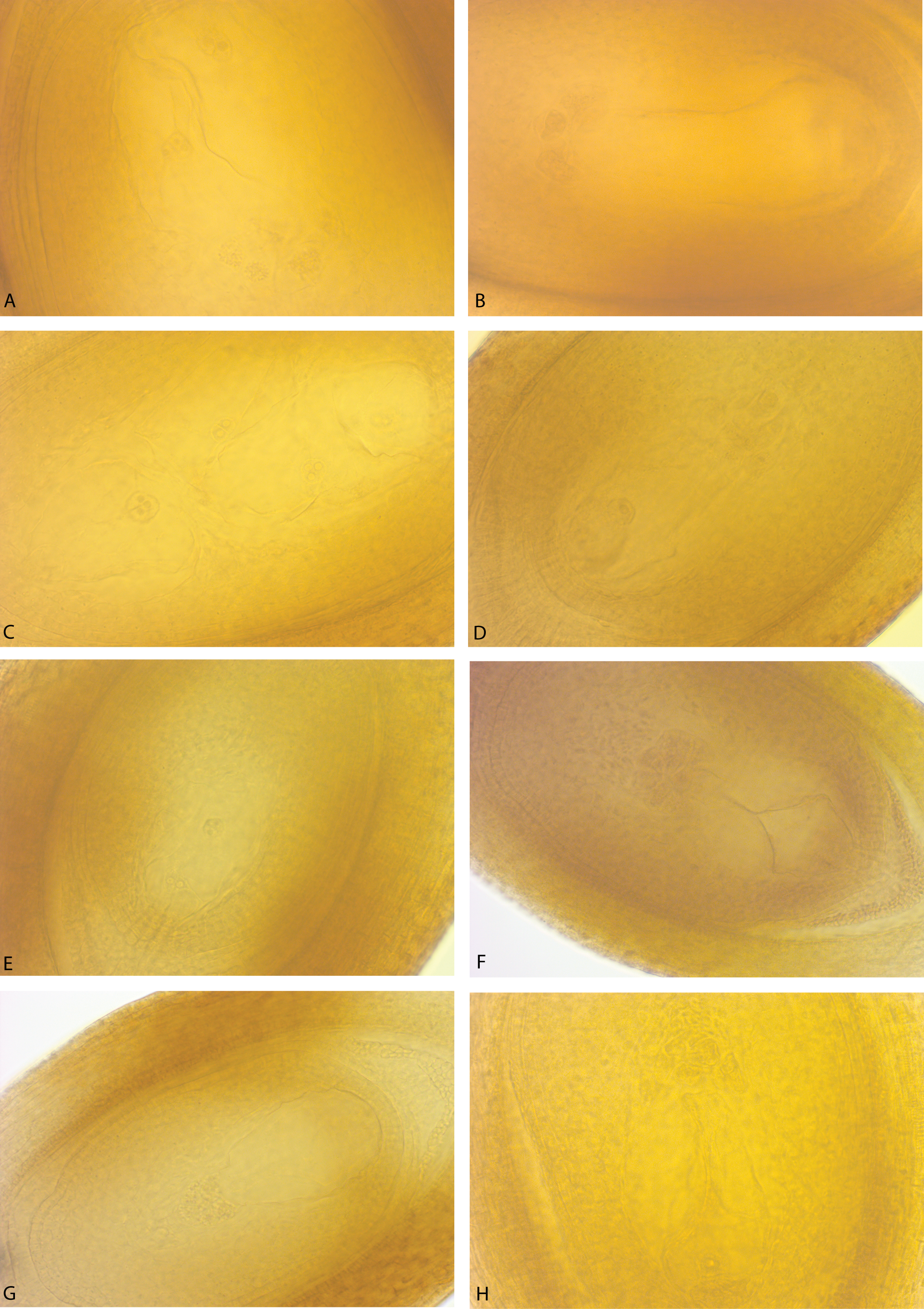

Supplement: Supplementary file 1 — Additional file 1: Figure S1. Clarified embryo sacs visualized by differential interference contrast (DIC) microscopy from the Paspalum notatum accessions evaluated in this work. (A) Apomictic and (B) sexual BGP 30 embryo sacs. (C) Apomictic and (D) sexual BGP 34 embryo sacs. (E) Apomictic embryo sac from BGP 115. (F), (G) and (H) Sexual embryo sacs from BGP 306, BGP 22 and BGP 216, respectively. [file 12864_2020_6518_MOESM1_ESM.png]

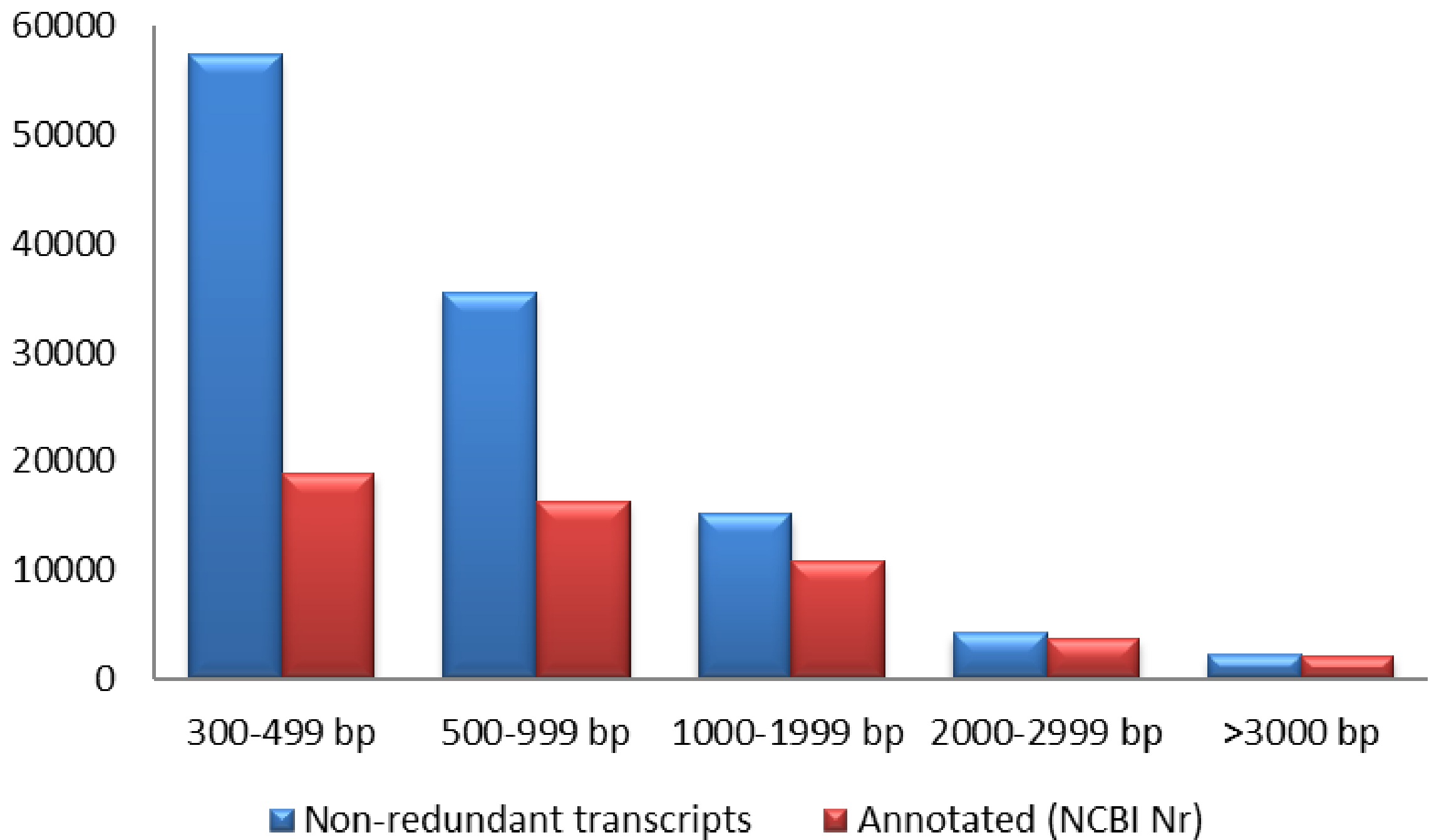

Supplement: Supplementary file 3 — Additional file 3: Figure S2. Length distribution of the number of nonredundant transcripts successfully annotated. Comparison of all nonredundant transcripts and the number of transcripts annotated in the NCBI nonredundant protein database by size range. [file 12864_2020_6518_MOESM3_ESM.pdf]

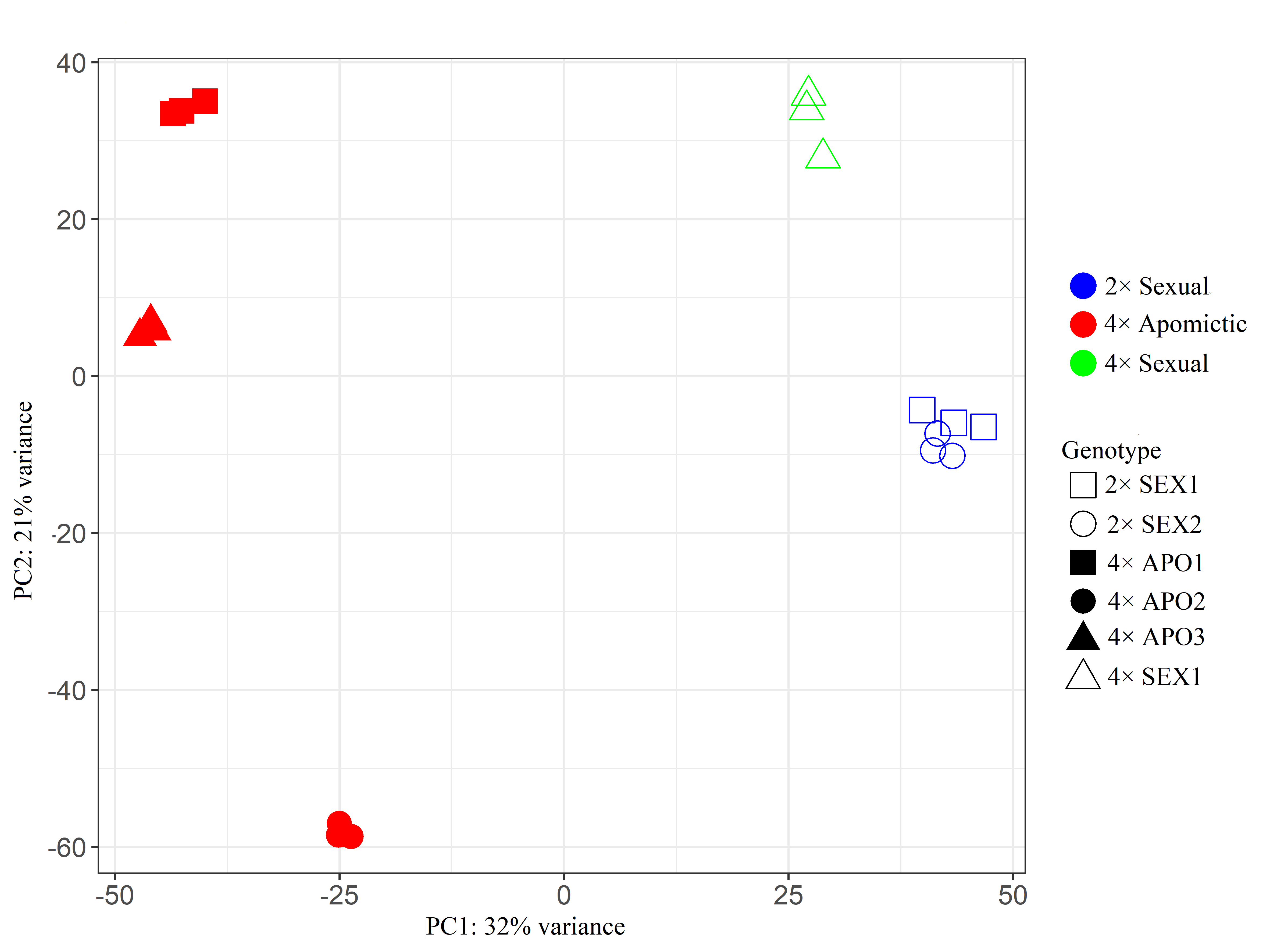

Supplement: Supplementary file 4 — Additional file 4: Figure S3. Principal component analysis (PCA) according to the FPKM values of the P. notatum transcriptome. PCA of leaf and floret transcriptomes of all genotypes and clones used in RNA-seq. [file 12864_2020_6518_MOESM4_ESM.tif]

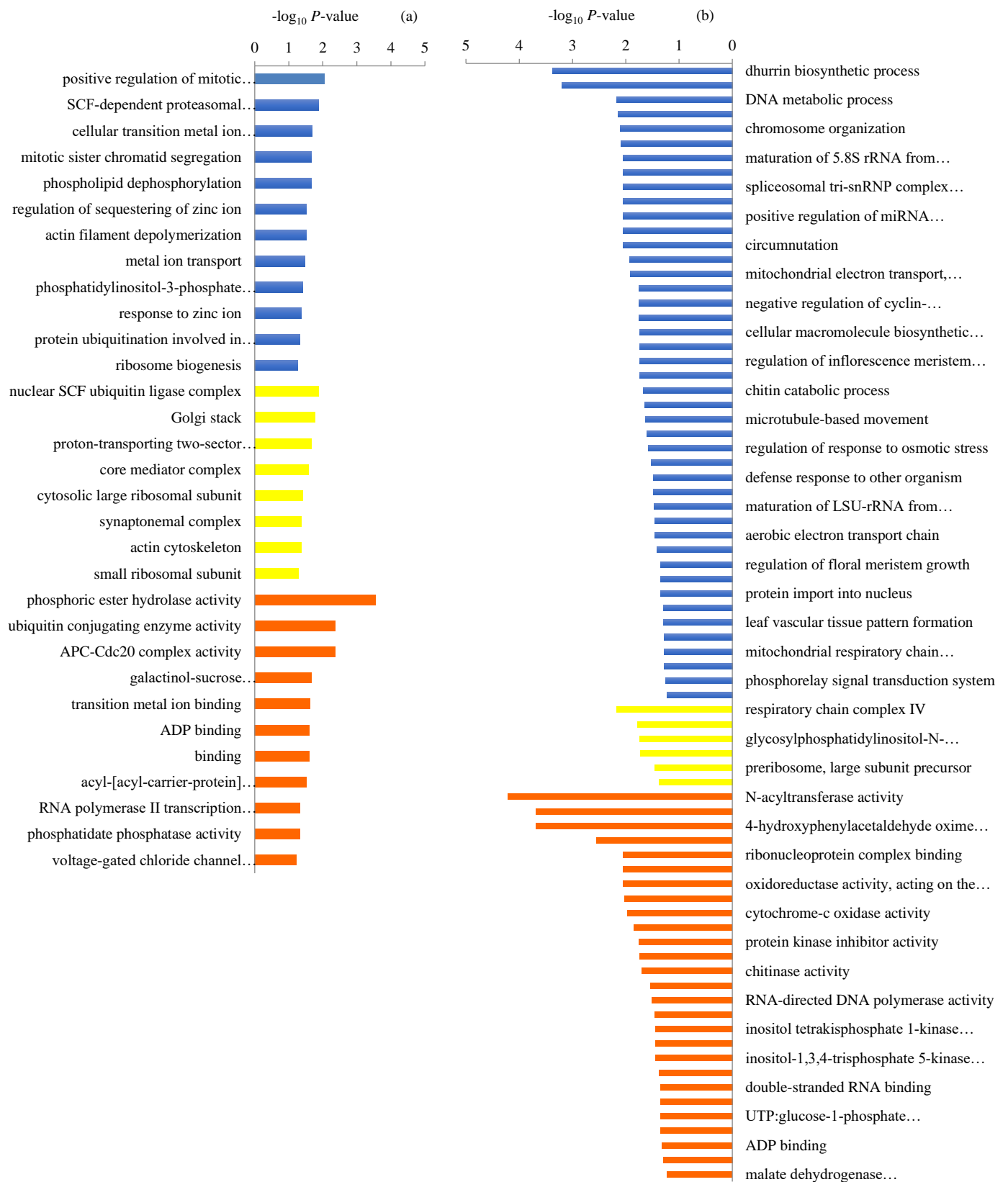

Supplement: Supplementary file 5 — Additional file 5: Figure S4. Functional classification of enriched overexpressed DEGs in the 2X sex vs. the 4X apo comparison. Gene Ontology biological process (blue boxes), Gene Ontology cellular component (yellow boxes), and Gene Ontology molecular function (orange boxes). a) Categories enriched in overexpressed transcripts in 2X sex and b) categories enriched in overexpressed transcripts in 4X apo. [file 12864_2020_6518_MOESM5_ESM.pdf]

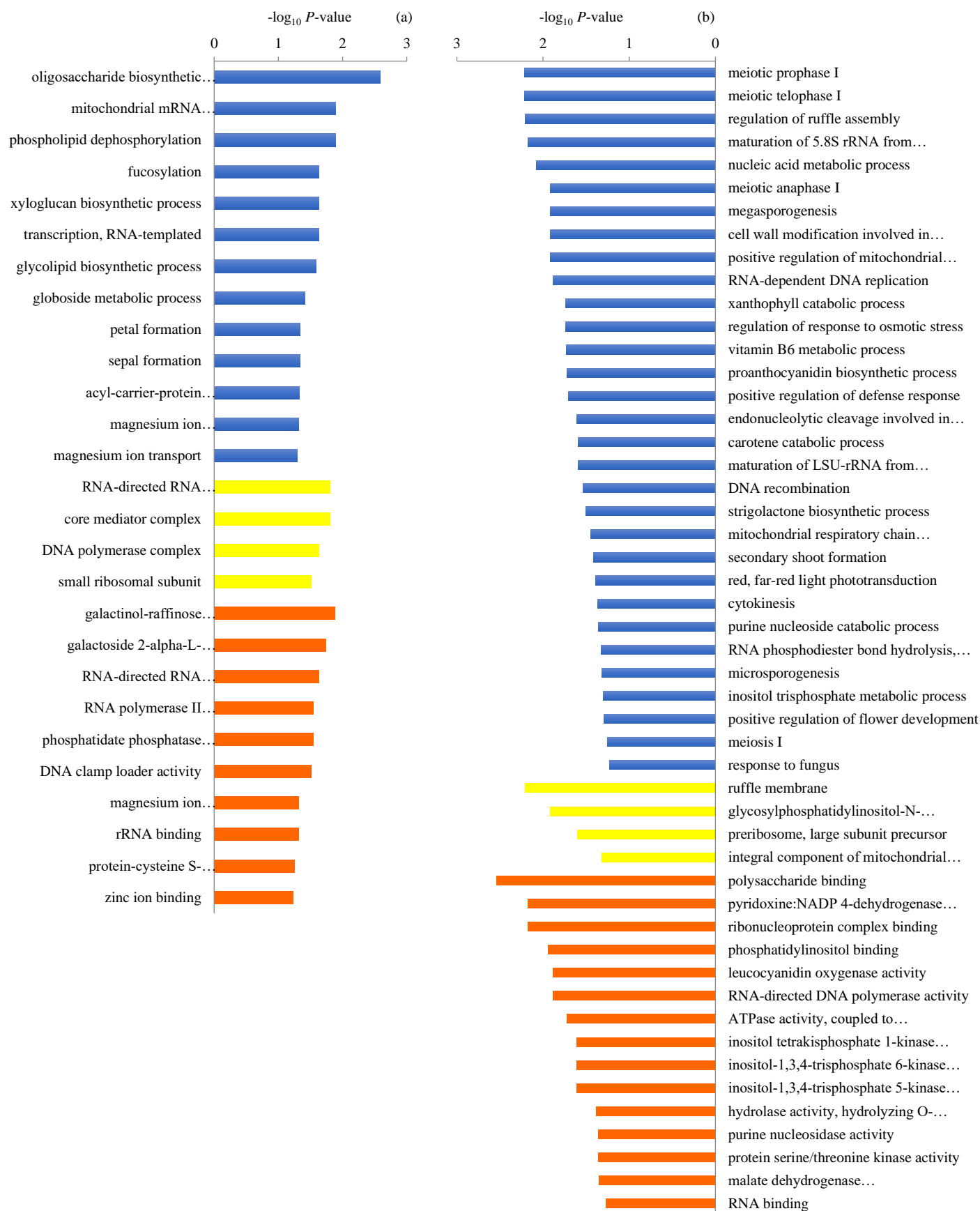

Supplement: Supplementary file 6 — Additional file 6: Figure S5. Functional classification of enriched overexpressed DEGs in the 2X sex vs. 4X sex comparison. Gene Ontology biological process (blue boxes), Gene Ontology cellular component (yellow boxes), and Gene Ontology molecular function (orange boxes). a) Categories enriched in overexpressed transcripts in 2X sex and b) categories enriched in overexpressed transcripts in 4X sex. [file 12864_2020_6518_MOESM6_ESM.pdf]

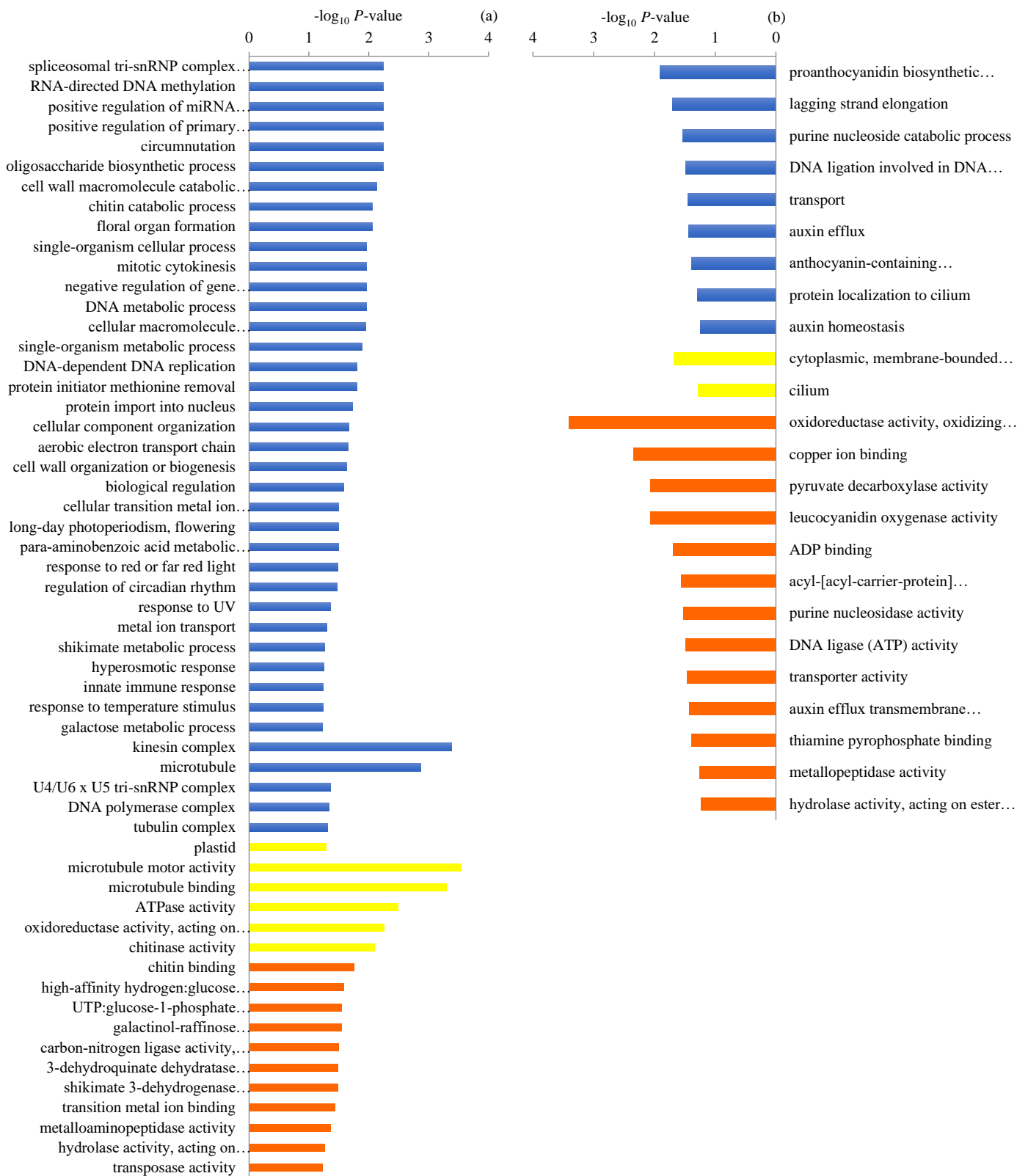

Supplement: Supplementary file 7 — Additional file 7: Figure S6. Functional classification of enriched overexpressed DEGs in the 4X apo vs. 4X sex comparison. Gene Ontology biological process (blue boxes), Gene Ontology cellular component (yellow boxes), and Gene Ontology molecular function (orange boxes). a) Categories enriched in overexpressed transcripts in 4X apo and b) categories enriched in overexpressed transcripts in 4X sex. [file 12864_2020_6518_MOESM7_ESM.pdf]

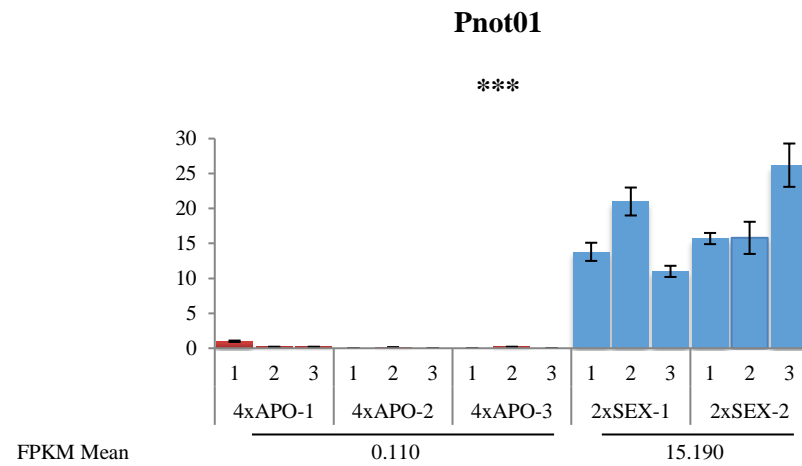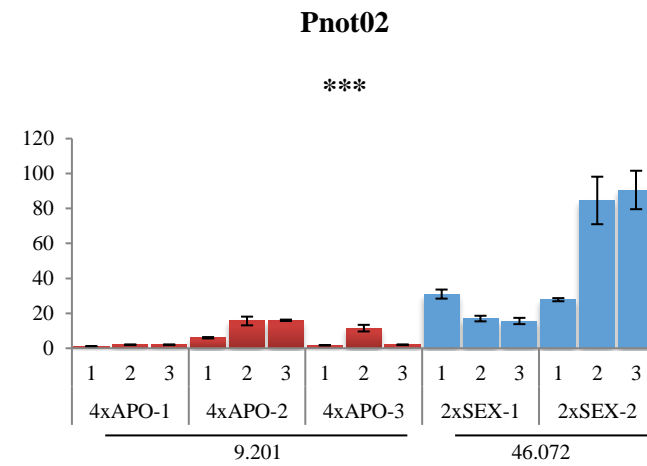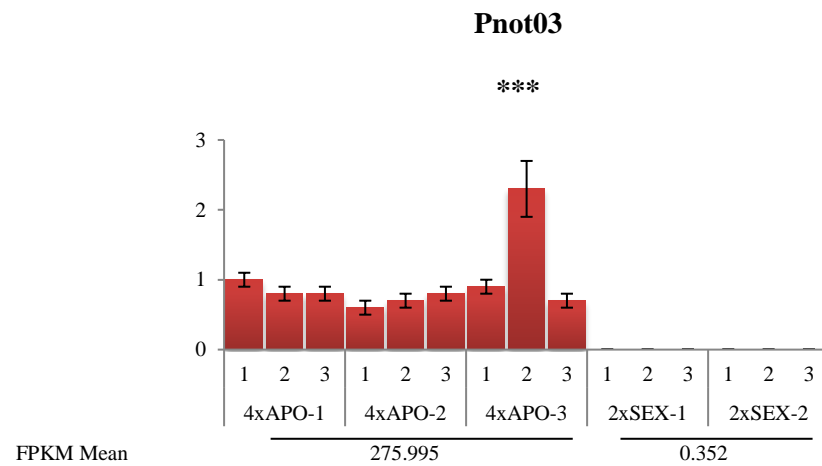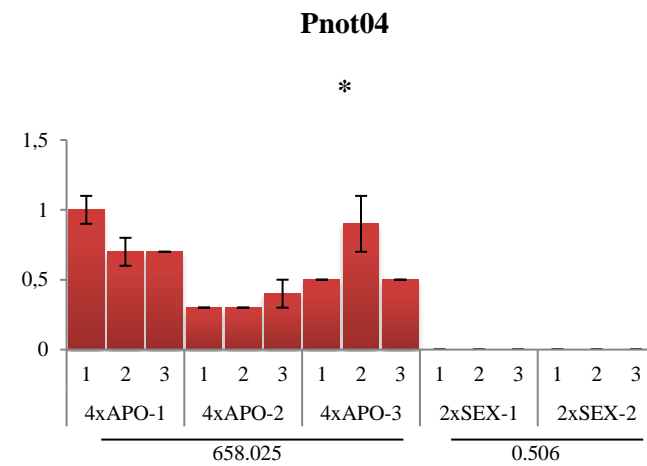

Supplement: Supplementary file 10 — Additional file 10: Figure S7. Histograms of gene expression obtained by qRT-PCR. qRT-PCR validation showing the relative expression patterns of 4 genes differentially expressed between the tetraploid apomictic “4X apo “ (red box) and diploid sexual “2 × SEX” (blue box) samples. *P < 0.05; **P < 0.01; ***P < 0.001; statistically significant differences in gene expression between phenotypic classes compared using the Mann-Whitney U-test. [file 12864_2020_6518_MOESM10_ESM.pdf]

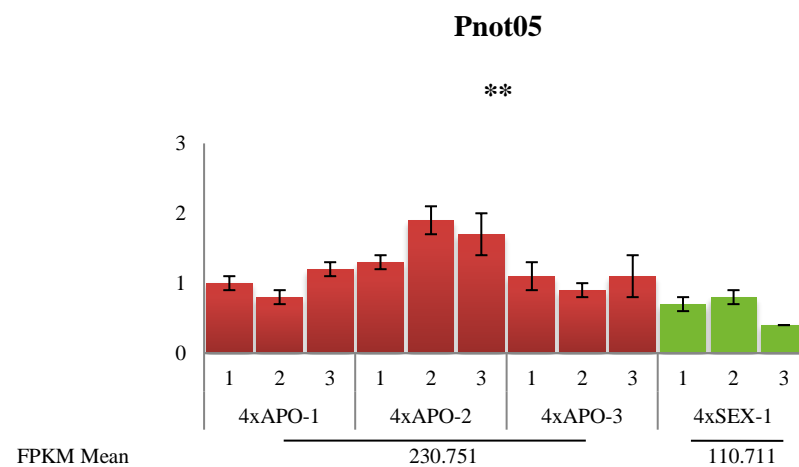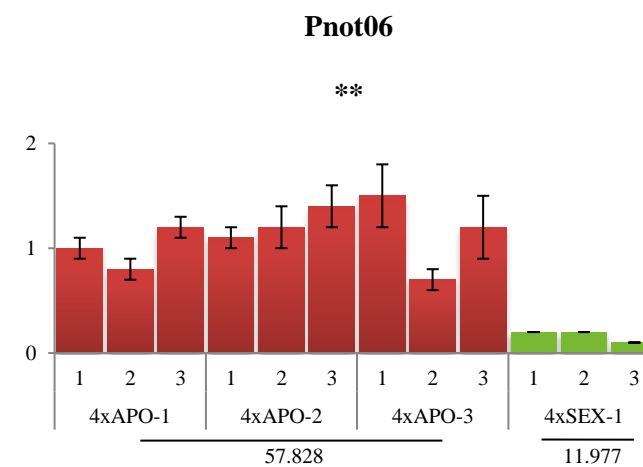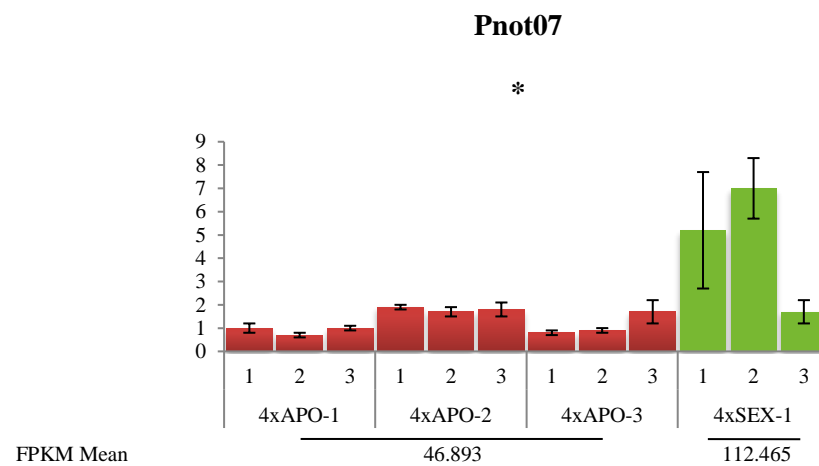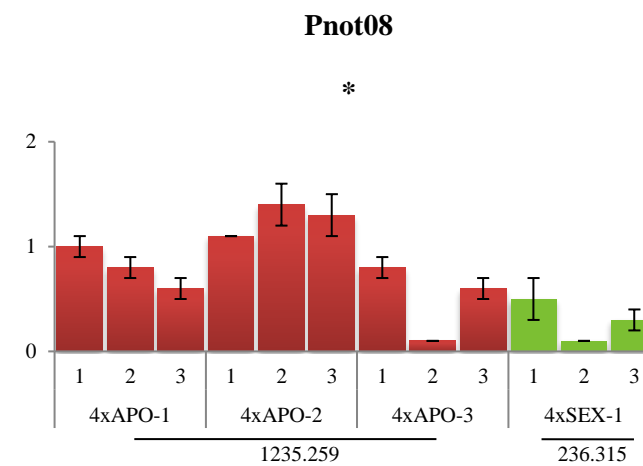

Supplement: Supplementary file 11 — Additional file 11: Figure S8. Histograms of gene expression obtained by qRT-PCR. qRT-PCR validation showing the relative expression patterns of 4 genes differentially expressed between the tetraploid apomictic “4X apo “ (red box) and tetraploid sexual “4X sex” (green box) samples. *P < 0.05; **P < 0.01; ***P < 0.001; statistically significant differences in gene expression between the phenotypic classes compared using the Mann-Whitney U-test. [file 12864_2020_6518_MOESM11_ESM.pdf]
