# Supplementary material for: Coexpression and Transcriptome analyses identify active Apomixis-related genes in Paspalum notatum leaves
Source: BMC Genomics. 2020 Jan 28;21:78. doi: 10.1186/s12864-020-6518-z (PMC6986084; doi:10.1186/s12864-020-6518-z)
Supplement: Supplementary file 2 — Additional file 2: Table S1. Statistics of the assembled transcriptome of Paspalum notatum. [file 12864_2020_6518_MOESM2_ESM.docx]

S1 Table. Statistics for the assembled transcriptome of *Paspalum notatum*.

|  | **Transcripts** | **Unigenes** |
| --- | --- | --- |
| Total | 203,808 | 114,306 |
| Total assembled bases | 219,683,637 | 85,787,466 |
| Average length (bp) | 1,077.90 | 750.51 |
| N50 length (bp) | 1,599 | 906 |
| GC percentage (%) | 46.76 | 47.60 |
