# Supplementary material for: Coexpression and Transcriptome analyses identify active Apomixis-related genes in Paspalum notatum leaves
Source: BMC Genomics. 2020 Jan 28;21:78. doi: 10.1186/s12864-020-6518-z (PMC6986084; doi:10.1186/s12864-020-6518-z)
Supplement: Supplementary file 8 — Additional file 8: Table S2. Primer sequences and amplicons of the candidate reference genes evaluated in this study. [file 12864_2020_6518_MOESM8_ESM.pdf]

ID	Primer Sequence (5’ – 3’)	Amplicon Length (bp)	Tm (°C)	Efficiency (%)	R^2	Gene Description / Annotation	E-value
Equally expressed genes between Diploid Sexual and Tetraploid Apomictic							
01RefGen-*Pnot*	F-TGCTTATGGACAGGGAGAGG<br>R-AGAAGGGGAAGGCAGAAAAA	128	60	95.3	0.99	40S ribosomal protein S13 (*Zea mays*)	1.1E-48
02RefGen-*Pnot*	F-GCAGTTCCTCTTGCTCTTGG<br>R-GCACAAACCATTGACACATCA	113	60	108.0	0.99	Uncharacterized protein (*Zea mays*)	1.2E-78
03RefGen-*Pnot*	F-TGGGACATCAGTGGTTGTTG<br>R-AGGTATTCTTTGAGCCATCGTG	80	60	92.7	0.98	Uncharacterized protein (*Setaria italica*)	140E-87

ID	Primer Sequence (5’ – 3’)	Amplicon Length (bp)	Tm (°C)	Efficiency (%)	R^2	Gene Description / Annotation	E-value
Equally expressed genes between Tetraploid Apomictic and Tetraploid Sexual							
04RefGen-*Pnot*	F-CAGGTGGCGGCTGAGTATT<br>R-AGGCGTTGGTTTTGACGAT	128	60	109.1	0.99	Putative uncharacterized protein Sb08g002920 (*Sorghum bicolor*)	24E-72
05RefGen-*Pnot*	F-CAGCCCCGAAATCCTTACTC<br>R-CCTCCACAATCACACATCCA	125	60	105.0	1.00	Putative uncharacterized protein Sb01g036850 (*Sorghum bicolor*)	16E-117
