# Supplementary material for: Coexpression and Transcriptome analyses identify active Apomixis-related genes in Paspalum notatum leaves
Source: BMC Genomics. 2020 Jan 28;21:78. doi: 10.1186/s12864-020-6518-z (PMC6986084; doi:10.1186/s12864-020-6518-z)
Supplement: Supplementary file 9 — Additional file 9: Table S3. Quantitative RT-PCR primer sequences. [file 12864_2020_6518_MOESM9_ESM.pdf]

ID	Primer Sequence (5' – 3')	Amplicon Length (bp)	RealFC	FPKM Mean		Annotation	E-value	GO terms
				2×SEX	4×APO			
Differentially expressed genes between Diploid Sexual and Tetraploid Apomictic								
Pnot01	F-CTGATCCAGTGCTTCGACTG<br>R-GTGCACATGGCCTCAAGA	110	127,045	15,190	0,110	Putative uncharacterized protein Sb02g007570 (Sorghum bicolor)	6.0E-33	P: defense response to other organism; F: iron ion binding; C: membrane; P: secondary metabolite biosynthetic process; F: oxidoreductase activity, acting on paired donors, with incorporation or reduction of molecular oxygen, NAD(P)H as one donor, and incorporation of one atom of oxygen; P: indole glucosinolate metabolic process; P: electron transport chain; F: heme binding
Pnot02	F-AGCAAGTCCTCCGCTCAC<br>R-TGCCTCTGCCTAAGTTCTCTC	123	5,003	46,072	9,201	Peroxidase (*Arundo donax*)	25E-111	P: plant-type cell wall organization; C: extracellular region; C: plant-type cell wall; F: heme binding; P: hydrogen peroxide catabolic process; F: peroxidase activity; P: response to oxidative stress; F: metal ion binding; P: obsolete peroxidase reaction; C: integral component of membrane; P: cellular oxidant detoxification; C: cytoplasmic, membrane-bounded vesicle; P: oxidation-reduction process
Pnot03	F-ACCACTCCCTATCCGTTCCT<br>R-TCCACATCGGCGTCATTAT	93	0,001	0,352	275,995	H0315A08.7 protein (*Oryza sativa*)	5.4E-54	F: ATP binding; P: stress-activated protein kinase signaling cascade; P: activation of protein kinase activity; P: regulation of apoptotic process; P: serine family amino acid metabolic process; F: protein serine/threonine kinase activity; P: signal transduction by protein phosphorylation; C: plastid; P: regulation of mitotic cell cycle
Pnot04	F-GGAGCAGAACCCAATGAGAC<br>R-GGGAAACACCAACTGTAATGG	100	0,001	0,506	658,025	Putative uncharacterized protein Sb03g008110 (*Sorghum bicolor*)	800E-156	——

ID	Primer Sequence (5' – 3')	Amplicon Length (bp)	RealFC	FPKM Mean		Annotation	E-value	GO terms
				4×APO	4×SEX			
Differentially expressed genes between Tetraploid Apomictic and Tetraploid Sexual								
Pnot05	F-TTGTGGAATGGAAGTGGAAA<br>R-TGTGGCTGTCAAAAAGGAACT	146	2,084	230,751	110,711	Uncharacterized protein (Setaria italica)	13E-78	P: reciprocal meiotic recombination; P: pollen development; P: chromatin silencing; P: telomere maintenance in response to DNA damage; P: post-translational protein modification; P:mitotic cell cycle; P: meiotic DNA double-strand break formation; P: multicellular organism reproduction; P: systemic acquired resistance; P:regulation of flower development; P: positive regulation of transcription, DNA-templated; P: histone phosphorylation; P: regulation of cell cycle; P: response to gamma radiation; P: histone H3-K9 methylation; P: negative regulation of systemic acquired resistance; P: regulation of telomere maintenance; P: protein deubiquitination; P: DNA replication initiation; P: sister chromatid cohesion; P: photomorphogenesis; P: male meiosis; P: double-strand break repair via homologous recombination; P: floral organ formation; P: regulation of DNA replication; P: DNA methylation; P: gene silencing by RNA; P: somatic cell DNA recombination; P: cullin deneddylation; P: protein ubiquitination
Pnot06	F-GGTGCCAAAACTCAGGAAA<br>R-TCCATCAACTGTCTCCATAGC	132	4,825	57,828	11,977	Uncharacterized protein (Setaria italica)	370E-81	P: DNA methylation; P: regulation of flower development; P: DNA-dependent DNA replication; C: plastid; P:histone H3-K9 methylation
Pnot07	F-GTGCTGCTTGGGCTTCTT<br>R-GAGCGTGAGCGACTACTGTG	70	0,417	46,893	112,465	Fiber Fb19	68E-33	P: response to stress
Pnot08	F-GAAAACGCCAATCTATGTCG<br>R-CATCCTCAAGCAGTTCCTCA	150	1,755	1235,259	236,315	Putative uncharacterized protein Sb08g018630 (Sorghum bicolor)	680E-174	P: chlorophyll biosynthetic process; P: L-phenylalanine biosynthetic process; P: jasmonic acid biosynthetic process; P: chloroplast organization; P: unsaturated fatty acid biosynthetic process; P: tryptophan biosynthetic process; P: positive regulation of transcription, DNA-templated; P: cysteine biosynthetic process; P: isopentenyl diphosphate biosynthetic process, methylerythritol 4-phosphate pathway; P: phosphorylation; P: indoleacetic acid biosynthetic process; P: tyrosine biosynthetic process; P: oxidoreduction coenzyme metabolic process; P: coenzyme biosynthetic process; P: leaf morphogenesis; P: tryptophan catabolic process; P: cell differentiation; P: thylakoid membrane organization; P: shikimate metabolic process; P: iron-sulfur cluster assembly
