# Supplementary material for: Coexpression and Transcriptome analyses identify active Apomixis-related genes in Paspalum notatum leaves
Source: BMC Genomics. 2020 Jan 28;21:78. doi: 10.1186/s12864-020-6518-z (PMC6986084; doi:10.1186/s12864-020-6518-z)
Supplement: Supplementary file 13 — Additional file 13: Table S5. Results of the BLAST search of sequences of P. notatum from the literature against the assembled transcriptome. [file 12864_2020_6518_MOESM13_ESM.pdf]

Query id	Subject id	Subject sequence origin	Subject start	Subject end	Percent %ID	Mismatches	Gaps	Alignment Length	Query Length	Query Coverage	E-value	Score
TR59083|c0_g1_i1	AF242537.1	Pessino et al., 2001	91.50	353	20	8	1248	1593	6	355	8,00E-137	477
TR59083|c0_g1_i1	AF242538.1		88.83	358	22	13	1248	1593	6	357	1,00E-120	424
TR59083|c0_g1_i1	AF242539.1		89.73	409	24	16	1248	1649	6	403	1,00E-145	507
TR35062|c2_g1_i1	GQ385196.1	Felitti et al., 2011	98.24	454	8	0	152	605	676	223	0.0	795
TR35062|c2_g2_i1	GQ385196.1		97.92	674	13	1	152	824	676	3	0.0	1166
TR35062|c2_g1_i1	GQ385197.1		98.49	530	7	1	1	529	532	3	0.0	933
TR35062|c2_g2_i1	GQ385197.1		98.49	530	7	1	1	529	532	3	0.0	933
TR35062|c2_g1_i1	GQ385198.1		98.30	529	9	0	1	529	531	3	0.0	928
TR35062|c2_g2_i1	GQ385198.1		98.30	529	9	0	1	529	531	3	0.0	928
TR35062|c2_g1_i1	GQ385199.1		99.55	445	2	0	85	529	445	1	0.0	811
TR35062|c2_g2_i1	GQ385199.1		99.55	445	2	0	85	529	445	1	0.0	811
TR35062|c2_g1_i1	GQ385200.1		98.99	493	5	0	37	529	495	3	0.0	883
TR35062|c2_g2_i1	GQ385200.1		98.99	493	5	0	37	529	495	3	0.0	883
TR35062|c2_g1_i1	GQ385201.1		97.36	530	13	1	1	529	532	3	0.0	900
TR35062|c2_g2_i1	GQ385201.1		97.36	530	13	1	1	529	532	3	0.0	900
TR35062|c2_g1_i1	GQ385202.1		99.43	529	2	1	1	529	528	1	0.0	959
TR35062|c2_g2_i1	GQ385202.1		99.43	529	2	1	1	529	528	1	0.0	959
TR35062|c2_g1_i1	GQ385203.1		99.77	444	1	0	86	529	446	3	0.0	815
TR35062|c2_g2_i1	GQ385203.1		99.77	444	1	0	86	529	446	3	0.0	815
TR35062|c2_g1_i1	GQ385204.1		100.00	255	0	0	152	406	257	3	2,00E-135	472
TR35062|c2_g2_i1	GQ385204.1		100.00	255	0	0	152	406	257	3	2,00E-135	472
TR35062|c2_g1_i1	GQ385205.1		99.74	378	1	0	152	529	380	3	0.0	693
TR35062|c2_g2_i1	GQ385205.1		99.74	378	1	0	152	529	380	3	0.0	693
TR5206|c0_g1_i1	KC792581.1	Podio et al., 2014	98.98	391	3	1	151	541	107	496	0.0	699
TR5206|c0_g1_i1	KC792581.1		94.63	242	8	3	1	240	51	289	4,00E-105	370
TR24308|c0_g1_i1	KC792581.1		99.15	351	1	2	1066	1414	1283	933	0.0	630
TR24308|c0_g1_i1	KC792581.1		97.79	272	6	0	796	1067	1738	1467	2,00E-134	470
TR24308|c0_g1_i1	KC792581.1		98.68	151	2	0	1402	1552	870	720	7,00E-74	268

TR24308|c0_g1_i1	KC792581.1		98.72	78	1	0	1551	1628	642	565	5,00E-35	139
TR24308|c0_g1_i1	KC792581.1		100.00	60	0	0	1626	1685	85	26	1,00E-26	111
TR874|c0_g1_i1	KC792582.2		98.99	493	5	0	1	493	1795	1303	0.0	883
TR874|c1_g1_i1	KC792582.2		99.20	375	3	0	142	516	985	1359	0.0	676
TR874|c1_g1_i1	KC792582.2		98.23	282	5	0	556	837	1772	2053	5,00E-142	494
TR874|c1_g1_i1	KC792582.2		98.62	145	0	1	1	143	733	877	3,00E-70	255
TR68788|c1_g1_i1	KC792582.2		99.63	273	1	0	1921	2193	533	805	2,00E-143	499
TR68788|c1_g1_i1	KC792582.2		99.23	130	1	0	1792	1921	323	452	7,00E-64	235
TR68788|c1_g1_i1	KC792582.2		100.00	120	0	0	1675	1794	117	236	5,00E-60	222
TR68788|c1_g2_i1	KC792582.2		81.12	286	52	2	243	527	1772	2056	4,00E-62	228
TR68788|c1_g3_i1	KC792582.2		95.00	120	6	0	586	705	117	236	2,00E-50	189
TR68788|c1_g3_i1	KC792582.2		100.00	32	0	0	703	734	323	354	1,00E-11	60.2
TR31014|c0_g1_i1	KM114904.1	Siena et al., 2014	100.00	40	0	0	361	400	1638	1677	3,00E-16	75.0
TR53162|c0_g1_i1	KM114904.1		95.88	972	27	5	347	1318	2174	1216	0.0	1561
TR67291|c0_g1_i1	KM114904.1		100.00	39	0	0	317	355	217	179	9,00E-16	73.1
TR31014|c0_g1_i1	KM114905.1		100.00	40	0	0	361	400	1656	1695	3,00E-16	75.0
TR53162|c0_g1_i1	KM114905.1		96.21	976	33	3	346	1318	2193	1219	0.0	1594
TR67291|c0_g1_i1	KM114905.1		100.00	39	0	0	317	355	217	179	9,00E-16	73.1
TR43425|c1_g1_i1	LN832398.1	Siena et al., 2016	91.60	643	48	2	176	815	2164	1525	0.0	883
TR55035|c0_g1_i1	LN832398.1		90.18	774	59	5	4	777	757	1	0.0	992
TR43425|c1_g1_i1	LN832399.1		91.60	643	48	2	176	815	2164	1525	0.0	883
TR55035|c0_g1_i1	LN832399.1		90.18	774	59	5	4	777	757	1	0.0	992
TR43425|c1_g1_i1	LN832400.1		93.12	640	37	4	176	815	2165	1533	0.0	931
TR55035|c0_g1_i1	LN832400.1		92.50	773	51	2	5	777	766	1	0.0	1099
TR43425|c1_g1_i1	LN832401.1		93.12	640	37	4	176	815	2165	1533	0.0	931
TR55035|c0_g1_i1	LN832401.1		92.50	773	51	2	5	777	766	1	0.0	1099
TR43425|c1_g1_i1	LN832402.1		93.75	640	40	0	176	815	2184	1545	0.0	961
TR55035|c0_g1_i1	LN832402.1		92.68	779	50	4	5	777	778	1	0.0	1116
TR43425|c1_g1_i1	LN832403.1		93.75	640	40	0	176	815	2184	1545	0.0	961
TR55035|c0_g1_i1	LN832403.1		92.68	779	50	4	5	777	778	1	0.0	1116
